# Butyrate Protects Mice Against Methionine–Choline-Deficient Diet-Induced Non-alcoholic Steatohepatitis by Improving Gut Barrier Function, Attenuating Inflammation and Reducing Endotoxin Levels

**DOI:** 10.3389/fmicb.2018.01967

**Published:** 2018-08-21

**Authors:** Jianzhong Ye, Longxian Lv, Wenrui Wu, Yating Li, Ding Shi, Daiqiong Fang, Feifei Guo, Huiyong Jiang, Ren Yan, Wanchun Ye, Lanjuan Li

**Affiliations:** ^1^State Key Laboratory for Diagnosis and Treatment of Infectious Diseases, The First Affiliated Hospital, School of Medicine, Zhejiang University, Hangzhou, China; ^2^Collaborative Innovation Center for Diagnosis and Treatment of Infectious Diseases, Hangzhou, China; ^3^Department of Infectious Disease, Shulan Hangzhou Hospital, Zhejiang University, Hangzhou, China; ^4^Department of Chemotherapy 2, Wenzhou Central Hospital, Wenzhou, China

**Keywords:** butyrate, microbiota, metabolome, methionine–choline-deficient diet, non-alcoholic steatohepatitis

## Abstract

Butyrate exerts protective effects against non-alcoholic steatohepatitis (NASH), but the underlying mechanisms are unclear. We aimed to investigate the role of butyrate-induced gut microbiota and metabolism in NASH development. Sixty-five C57BL/6J mice were divided into four groups (*n* = 15–17 per group) and were fed either a methionine–choline-sufficient (MCS) diet or methionine–choline-deficient (MCD) diet with or without sodium butyrate (SoB; 0.6 g/kg body weight) supplementation for 6 weeks. Liver injury, systematic inflammation, and gut barrier function were determined. Fecal microbiome and metabolome were analyzed using 16S rRNA deep sequencing and gas chromatography-mass spectrometry (GC-MS). The results showed that butyrate alleviated the MCD diet-induced microbiome dysbiosis, as evidenced by a significantly clustered configuration separate from that of the MCD group and by the depletion of *Bilophila* and *Rikenellaceae* and enrichment of promising probiotic genera *Akkermansia*, *Roseburia*, *Coprococcus*, *Coprobacillus*, *Delftia*, *Sutterella*, and *Coriobacteriaceae* genera. The fecal metabolomic profile was also substantially improved by butyrate; several butyrate-responsive metabolites involved in lipid metabolism and other pathways, such as stearic acid, behenic acid, oleic acid, linoleic acid, squalene, and arachidonic acid, were identified. Correlation analysis of the interaction matrix indicated that the modified gut microbiota and fecal metabolites induced by butyrate were strongly correlated with the alleviation of hepatic injury, fibrosis progression, inflammation, and lipid metabolism and intestinal barrier dysfunction. In conclusion, our results demonstrated that butyrate exerts protective effects against NASH development, and these effects may be driven by the protective gut microbiome and metabolome induced by butyrate. This study thus provides new insights into NASH prevention.

## Introduction

The prevalence of non-alcoholic fatty liver disease (NAFLD), a major public health concern, has increased worldwide, and this disease is commonly associated with obesity, insulin resistance, diabetes, and the metabolic syndrome (Rosso et al., 2016). NAFLD encompasses a pathological spectrum ranging from isolated steatosis to non-alcoholic steatohepatitis (NASH), which can eventually progress to fibrosis, cirrhosis or hepatocellular carcinoma (Le Roy et al., 2013).

A combination of genetic, metabolic, lifestyle, and environmental factors substantially contribute to the pathogenesis of NAFLD. However, despite extensive studies conducted in recent decades, the underlying molecular mechanisms of NAFLD progression remain mostly unclear; common treatment strategies, other than modifiable lifestyle factors such as weight loss diets and/or intensive physical activity are not yet available, and no effective drug for NASH treatment has been approved by the Food and Drug Administration (Jin et al., 2015; Loomba et al., 2015).

Increasingly, studies have confirmed the important role of the gut microbiota in NAFLD development and progression, but the underlying mechanisms remain unclear (Le Roy et al., 2013). Endogenous short-chain fatty acids (SCFAs) are mainly produced from dietary carbohydrates by the gut microbiota, composed of many intestinal commensal bacteria and/or probiotic bacteria (Ye et al., 2017). Interestingly, dietary supplementation with SCFAs, specifically butyrate, has been shown to protect against high-fat diet (HFD)-induced obesity, insulin resistance and hepatic steatosis in animal models (Mattace Raso et al., 2013; Zhou et al., 2017). However, the ability of butyrate to protect mice from methionine–choline-deficient (MCD) diet-induced NASH development and to modulate the gut microbiota and gut luminal metabolism has not yet been determined. We hypothesized that butyrate can attenuate liver steatosis and liver injury and improve gut microbiota and gut luminal metabolism in a mouse model of NASH induced by an MCD diet.

## Materials and Methods

### Ethics Statement

The animal protocol was approved by the Animal Care Committee of Zhejiang University School of Medicine (permit number: 2017-591) and performed in accordance with the “Guidelines for Experimental Animals” of the Ministry of Science and Technology (Beijing, China).

### Animals and Treatments

Eight-week-old male C57BL/6J mice were housed in a laminar flow, specific pathogen-free facility. After their arrival, the mice were acclimatized for 1 week with free access to a standard chow diet and water. Then, the mice were randomly divided into the following four groups (*n* = 15–17/group) for a 6-week-long feeding period: (1) One group was fed a methionine–choline-sufficient (MCS) diet (Research Diet, New Brunswick, NJ, United States) and was treated by gavage with 0.12 g/ml of sodium butyrate (SoB) (Sigma-Aldrich, St. Louis, MO, United States) (Control + SoB group, 0.6 g/kg body weight). (2) One group was fed an MCD diet (Research Diet, New Brunswick, NJ, United States) and was treated by gavage with 0.12 g/ml of SoB (MCD + SoB group, 0.6 g/kg body weight). (3) The Control group received the MCS diet and vehicle administered by gavage. (4) The MCD group received the MCD diet and vehicle administered by gavage. The specifications of the MCD and MCS diets are summarized in **Supplementary Table S1**. After 6 weeks, the mice were euthanized by intraperitoneal injection of 4% chloral hydrate (with 1 mg/100 ml atropine to inhibit respiratory secretions) for tissue collection.

### Sample Collection

Fecal samples were collected from all mice upon defecation at the end point (week 6) and were stored in a -80°C freezer for the subsequent analyses. Blood samples were collected immediately after the mice were anesthetized and were centrifuged at 3,000 rpm for 15 min; all serum aliquots were kept at -80°C. Liver and colon samples were either fixed in 4% neutral-buffered formaldehyde and embedded in paraffin or in Allprotect Tissue Reagent (Qiagen, Valencia, CA, United States) or snap-frozen in liquid nitrogen and stored at -80°C.

### Histological Evaluation of Liver Tissues and Triglycerides Content

Paraffin-embedded liver sections were stained with hematoxylin–eosin (HE) or Masson’s trichrome to detect liver injury and fibrosis and were scanned using a NanoZoomer Digital Pathology system (Hamamatsu Photonics, K.K., Japan), which digitally scans the sections into a specific image format for further evaluation. The HE-stained sections were scored using the NAFLD activity score (NAS) system (Kleiner et al., 2005). Fibrosis was analyzed and quantified using Image-Pro Plus software (version 6.0, Media Cybernetics, Rockville, MD, United States). For each section, the blue regions (collagen) were normalized to the red regions (hepatocytes). The fibrosis index (%) was calculated as a percentage of the total tissue area and represented the average of six randomly selected fields (20× magnification) from each section. Liver triglycerides (TG) levels were determined using a TG assay kit (Applygen Technologies Inc., Beijing, China) according to manufacturer’s protocols. Final concentrations were normalized to the corresponding protein content.

### Serum Parameter Analysis

Alanine aminotransferase (ALT) and aspartate aminotransferase (AST) levels were measured using a dry chemistry analyzer (FUJI DRI-CHEM 7000V, FUJIFILM, Tokyo, Japan). To indirectly quantify endotoxin levels, lipopolysaccharide (LPS)-binding protein (LBP) concentrations were measured by ELISA according to the manufacturer’s guidelines (Guduo, Shanghai, China) because of the well-documented inaccuracies inherent to direct endotoxin measurements (Tremellen et al., 2017). Serum cytokine levels were measured using a Bio-Plex Mouse Cytokine 23-Plex Panel kit (Bio-Rad, Hercules, CA, United States) according to the manufacturer’s instructions. The following mouse cytokine levels were analyzed for each sample: interleukin (IL)-1α, IL-1β, IL-2, IL-3, IL-4, IL-5, IL-6, IL-9, IL-10, IL-12 p40, IL-12 p70, IL-13, IL-17α, eotaxin, granulocyte colony-stimulating factor (G-CSF), granulocyte-macrophage colony-stimulating factor (GM-CSF), gamma interferon (IFN-γ), keratinocyte-derived chemokine (KDC), macrophage chemoattractant protein 1 (MCP)-1, macrophage inflammatory protein-1α (MIP)-1α, MIP-1β, regulated upon activation normal T-cell expressed and secreted (RANTES), and tumor necrosis factor (TNF)-α.

### Immunohistochemical and Immunofluorescence Staining

Paraffin-embedded liver sections were stained for F4/80 (a marker of active macrophages) and alpha smooth muscle actin (α-SMA) (a hallmark of fibrosis) with standard immunohistochemical (IHC) staining procedures as previously detailed (Keren-Shaul et al., 2017). Briefly, liver sections were incubated with a specific primary antibody (Abcam PLC, Cambridge, United Kingdom), followed by incubation with an HRP-linked secondary antibody (Dako, Glostrup, Denmark) and 3,3′-diaminobenzidine (Dako, Glostrup, Denmark) and were scanned using the NanoZoomer Digital Pathology system. F4/80^+^ cells were counted using Image-Pro Plus. Quantitative analysis of α-SMA was performed using Image-Pro Plus software as previously described (Julio Junior et al., 2017). Six fields (20× magnification) of view were randomly selected on each section. The mean optical density of the images was considered the representative staining intensity of the target protein. Likewise, paraffin-embedded colon sections were stained for ZO-1 (a marker of the intestinal barrier) with standard immunofluorescence staining procedures as previously described (Chung et al., 2014). In brief, colon sections were incubated with rabbit polyclonal ZO-1 antibody (Proteintech, Rosemont, IL, United States), followed by incubation with Texas Red-conjugated goat anti-rabbit antibody (Jackson, PA, United States) and 4′,6-diamino-2-phenyl indole (DAPI) (Sigma-Aldrich, St. Louis, MO, United States) and were visualized with a Zeiss LSM T-PMT confocal microscope (Zeiss, Jena, Germany).

### RNA Extraction and Real-Time PCR Analysis

Total liver and colon tissue RNA was extracted using an RNeasy Plus Mini kit (Qiagen, Valencia, CA, United States) according to the manufacturer’s protocols. Real-time PCR was performed with an Applied Biosystems ViiA7 Real-time PCR system using a one-step SYBR PrimeScript plus RT-PCR kit (Takara Biomedicals, Kusatsu, Japan). The PCR primer sequences are listed in **Supplementary Table S2**. Samples were tested in duplicate in a 96-well plate. The expression data were normalized to the expression of the internal control GAPDH and were calculated according to the ΔΔCT method.

### Fecal SCFA Concentration Detection

Fecal SCFA concentration was detected by ELISA according to the manufacturer’s guidelines (Jianglai, Shanghai, China). The data were normalized to the total weight of feces.

### Microbial Community Analysis in Fecal Samples

DNA extraction from fecal samples was conducted using a QIAamp Fast DNA Stool Mini Kit (Qiagen, Valencia, CA, United States) following the manufacturer’s protocols. DNA concentration and integrity were measured by a NanoDrop 2000 spectrophotometer (Thermo Fisher Scientific, Hudson, NH, United States) and agarose gel electrophoresis, respectively. The library for 16S rDNA amplicon sequencing was constructed based on the PCR-amplified V3–V4 variable regions. The amplicon was purified using Agencourt AMPure XP beads (Beckman Coulter, Inc., Brea, CA, United States). The qualified libraries were paired-end sequenced with an Illumina MiSeq platform according to manufacturer’s protocols. Raw sequencing data were subjected to filtration using Trimmomatic, FLASH, and QIIME software. Then, clean reads were clustered into operational taxonomic units (OTUs) using UPARSE software with a 97% threshold. A representative read from each OTU was selected with the QIIME package (Caporaso et al., 2010). Representative OTU sequences were annotated and taxonomically classified using the Ribosomal Database Project (RDP) Classifier v.2.2, which had been aligned with the Silva database version 123 (Cole et al., 2009). The linear discriminant analysis (LDA) effect size (LEfSe) method^1^ was used to identify taxa with statistical significance and biological relevance (Lu et al., 2016).

### Metabolomic Profiling of Fecal Samples

Metabolomic profiling was performed as previously described (Liu et al., 2017) with some modifications. Briefly, fecal metabolites were extracted by mixing 15 mg of feces with 800 μl of ice-cold methanol (Sigma-Aldrich, St. Louis, MO, United States). After homogenization and centrifugation, the supernatant was transferred into an Eppendorf tube containing 20 μl of 1 mg/ml heptadecanoic acid (Sigma-Aldrich, St. Louis, MO, United States) as the internal standard; then, the sample was dried using a nitrogen stream (Aosheng, Hangzhou, China). The residue was reconstituted in 50 μl of 15 mg/ml methoxylamine hydrochloride (Sigma-Aldrich, St. Louis, MO, United States) in anhydrous pyridine (Sigma-Aldrich, St. Louis, MO, United States) and was incubated at 37°C for 24 h. Then, 50 μl of *N*,*O*-bistrifluoroacetamide (BSTFA) [with 1% trimethylsilyl chloride (TMCS)] (Sigma-Aldrich, St. Louis, MO, United States) was added to the mixture, and the sample was incubated at 70°C for 120 min. Metabolomic analysis was performed with gas chromatography-mass spectrometry (GC-MS) on an Agilent 7890A GC system coupled to an Agilent 5975C inert mass selective detector (MSD) system (Agilent Technologies, Santa Clara, CA, United States). For data analysis, ChemStation software (version E.02.02.1431, Agilent, Santa Clara, CA, United States) and ChromaTOF software (version 4.34, LECO, St Joseph, MI, United States) were used. Metabolites were identified by the NIST and Fiehn databases. Principal component analysis (PCA) and orthogonal partial least squares-discriminant analysis (OPLS-DA) were performed to visualize the metabolic differences among the experimental groups. Differential metabolites were selected according to the statistically significant variable importance in the projection (VIP) values obtained from the OPLS-DA model and P values from two-tailed Student’s *t*-tests on the normalized peak areas; metabolites with VIP values >1 and P values <0.05 were included.

### Statistical Analysis

The data are presented as the means ± SEM, and the Kolmogorov–Smirnov test was used to check for normality. For most data, one-way ANOVA with Tukey’s *post hoc* test was used to determine the significance between the groups. The Wilcoxon rank sum test was performed to evaluate alpha diversity and principal coordinates between the different cohorts in the 16S sequencing analysis. PERMANOVA (Adonis) was used to test for microbial community clustering using weighted and unweighted UniFrac distance matrices and Bray–Curtis distance matrices. Correlations between variables were computed using Spearman’s rank correlation. *P*-values < 0.05 were considered significant. The data were analyzed using SPSS version 17.0 for Windows (SPSS Inc., Chicago, IL, United States).

## Results

### Butyrate Alleviated Hepatic Injury and Inflammation and Improved Lipid Metabolism

**FIGURE 1:**
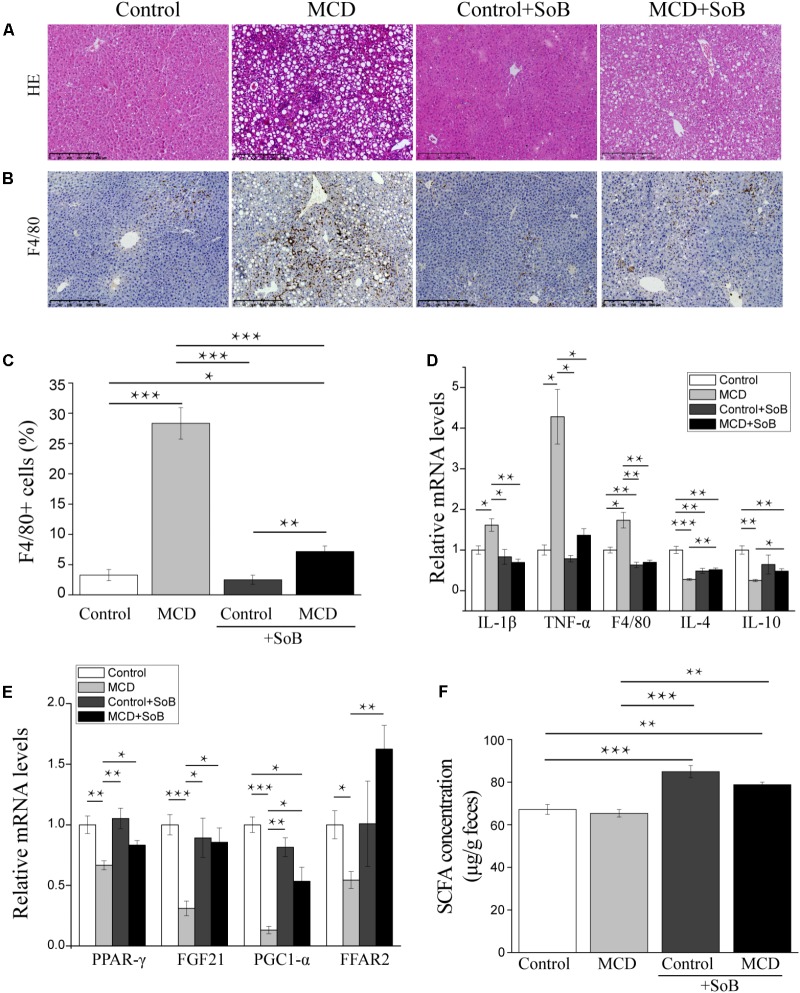
Butyrate alleviated hepatic injury and inflammation and regulated the expression of lipid metabolism-related genes. **(A,B)** Representative liver histology assessed by HE staining **(A)** and F4/80 (macrophage marker) staining **(B)**. Scale bar: 250 μm. **(C)** Percentage of F4/80^+^ positive cells. **(D,E)** Relative mRNA levels of inflammation-associated genes **(D)** and lipid metabolism-associated genes **(E)**. **(F)** Fecal SCFA concentration (μg/g feces). Data are given as the means ± SEM. *N* = 15–17 per group. ^∗^*P* < 0.05, ^∗∗^*P* < 0.01, and ^∗∗∗^*P* < 0.001 by one-way ANOVA with *post hoc* Tukey’s test.

As expected, the liver sections from mice fed the MCD diet showed extensive macrovesicular fat accumulation, which is associated with mixed inflammatory infiltration (**Figure 1A**). Similarly, the evaluation of liver damage using the NAS system demonstrated major hepatic steatosis with lobular inflammation and ballooning hepatocytes in MCD diet-fed mice (**Table 1**). In contrast, the mice fed the MCD diet supplemented with SoB (the MCD + SoB group) predominantly developed microvesicular steatosis and had a significantly lower number of inflammatory focus in the liver (**Figure 1A**). These effects were also associated with a reduction in the NAS score (**Table 1**), but fat accumulation in the MCD + SoB group was significantly higher than that in the Control group or the Control + SoB group (**Figure 1A** and **Table 1**). After 6 weeks, compared with the Control group, the MCD group displayed significantly increased serum levels of ALT (*P* < 0.01), AST (*P* < 0.01), and intrahepatic TG (*P* < 0.001) (**Table 1**), but compared to those in the MCD group, these levels were significantly reduced in the MCD + SoB group (*P* < 0.05, *P* < 0.01, and *P* < 0.05, respectively; **Table 1**).

**Table 1:** Effects of butyrate supplementation on the NAS and ALT, AST, and TG levels in the liver and LBP levels in the serum^1^.

	Control	MCD	Control + SoB	MCD + SoB
NAFLD activity score	0.29 ± 0.11	4.18 ± 0.21***	0.21 ± 0.11###	2.53 ± 0.24***###
Steatosis	0.29 ± 0.11	2.29 ± 0.11***	0.21 ± 0.11###	1.47 ± 0.19***##
Inflammation	0.00 ± 0.00	1.12 ± 0.08***	0.00 ± 0.00###	0.33 ± 0.13###
Ballooning	0.00 ± 0.00	0.76 ± 0.18**	0.00 ± 0.00##	0.73 ± 0.23*
Alanine aminotransferase (U/L)	19.29 ± 0.64	332.25 ± 26.55**	19.69 ± 1.51##	137.13 ± 11.33***#
Aspartate aminotransferase (U/L)	91.93 ± 2.98	400.25 ± 25.05**	111.31 ± 5.39*##	240.38 ± 19.68**##
Triglyceride (nmol/mg protein)	277.86 ± 32.15	574.89 ± 29.39***	326.98 ± 41.95###	460.26 ± 35.25**#
Lipopolysaccharide binding protein (μmol/L)	329.43 ± 13.13	398.82 ± 7.84***	317.81 ± 11.74###	321.67 ± 9.31###


*^*1*^Data are presented as the means ± SEM of 15–17 mice per group. ^∗^P < 0.05, ^∗∗^P < 0.01, ^∗∗∗^P < 0.001 compared with mice fed with the control diet. ^#^P < 0.05, ^##^P < 0.01, ^###^P < 0.001 compared with mice fed with the MCD diet.*

The mRNA expression levels of IL-1β and TNF-α were significantly higher in the livers of mice in the MCD group than those in the livers of mice in the Control group (*P* < 0.05 and *P* < 0.05, respectively) or the Control + SoB group (*P* < 0.05 and *P* < 0.05, respectively) but were normalized by butyrate treatments (**Figure 1D**). Similarly, the mRNA expression of F4/80, a specific marker of macrophage infiltration (Mattace Raso et al., 2013), was significantly increased in the MCD diet-fed mice compared to that in the control mice (*P* < 0.05) and was reduced by butyrate supplementation (*P* < 0.001; **Figure 1D**). Consistent with this finding, IHC analysis indicated that the infiltration of F4/80^+^ cells was significantly reduced in the livers of mice fed the MCD diet supplemented with SoB compared with that in the livers of mice fed the MCD diet alone (*P* < 0.01; **Figures 1B,C**). Moreover, the expression of IL-4 and IL-10, two well-known anti-inflammatory cytokines (Zhou et al., 2017), were downregulated in mice fed the MCD diet but were significantly up-regulated in mice concomitantly fed butyrate with the MCD diet (*P* < 0.01 and *P* < 0.05, respectively; **Figure 1D**). Consistent with these inflammatory modifications, lipid metabolism was impaired in the MCD diet-fed mice. The expression of peroxisome proliferator activated-receptor (PPAR-γ), which promotes fatty acid uptake and increases insulin sensitivity (Gurnell, 2003), was decreased in the livers of the mice fed the MCD diet (*P* < 0.01), and this reduction was significantly prevented by butyrate supplementation (*P* < 0.05; **Figure 1E**). Similarly, the expression of fibroblast growth factor (FGF)-21, which regulates glucose and lipid metabolism via pleiotropic actions (Li et al., 2012), was significantly downregulated by the MCD diet (*P* < 0.001) and was restored by butyrate supplementation (*P* < 0.05; **Figure 1E**). The mRNA expression of the PPAR-γ, the coactivator PGC1-α, was also significantly reduced in the MCD group (*P* < 0.001), and butyrate treatment prevented this change (*P* < 0.05; **Figure 1E**). Free fatty acid receptor (FFAR2) was also involved in the observed metabolic impairment. As shown in **Figure 1E**, the MCD diet induced a significant decrease in hepatic FFAR2 mRNA expression (*P* < 0.05), and SoB supplementation significantly increased FFAR2 transcription (*P* < 0.01).

To validate whether butyrate administration affected the physiological relevance of the model, we measured total SCFA concentrations. As shown in **Figure 1F**, compared to those in the MCD and Control groups, SCFA concentrations were significantly increased in the MCD + SoB and Control + SoB groups.

### Butyrate Ameliorated Liver Fibrosis Progression and Regulated Toll-Like Receptors

**FIGURE 2:**
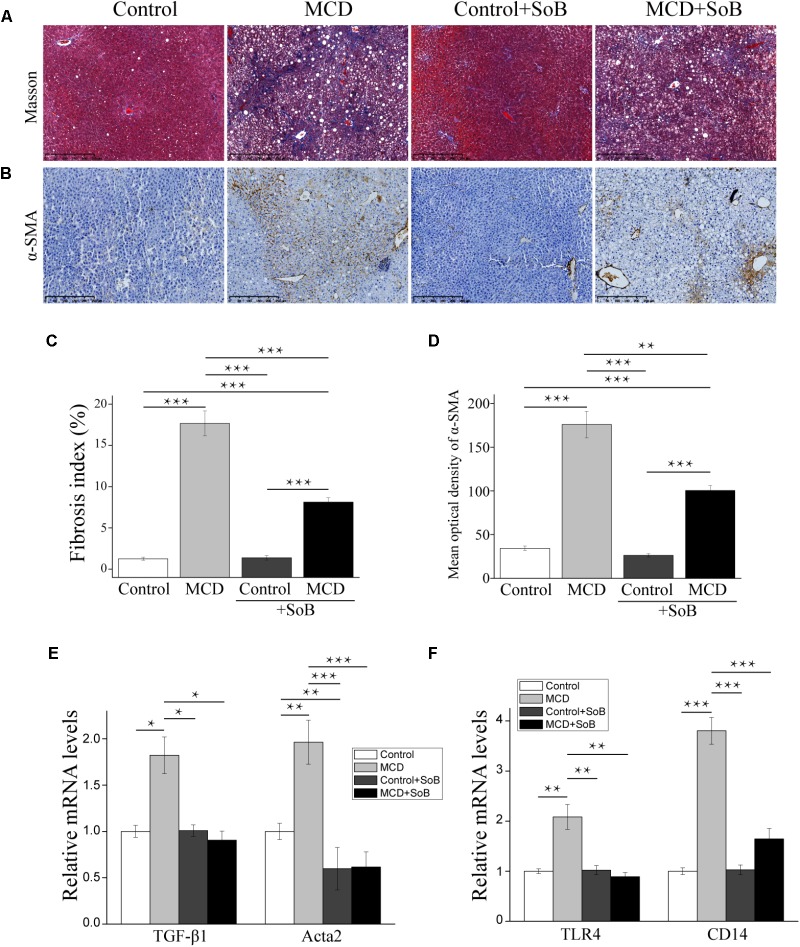
Butyrate ameliorated liver fibrosis progression and regulated toll-like receptors. **(A,B)** Representative liver histology assessed by Masson’s trichrome staining **(A)** and α-SMA staining **(B)**. Scale bar: 250 μm. **(C)** Percentage of Masson’s trichrome-stained area indicative of the fibrosis index (%). **(D)** Representative staining intensities for α-SMA as designated by the mean optical density. **(E,F)** Gene expression levels of the fibrosis marker TGF-β1, α-SMA encoding gene acta2 **(E)** and TLR4 and its co-receptor CD14 **(F)**. Data are presented as the means ± SEM. *N* = 15–17 per group. ^∗^*P* < 0.05, ^∗∗^*P* < 0.01, and ^∗∗∗^*P* < 0.001 by one-way ANOVA with *post hoc* Tukey’s test.

Fibrosis progression in the liver was evaluated using Masson’s trichrome staining, which stains collagen fibers blue. According to **Figure 2A**, mice in the Control group displayed with a normal lobular structure with veins and radial hepatic cords in the liver. However, mice in the MCD group displayed with signs of fibrosis including periportal and interstitial collagen deposition. The MCD + SoB group displayed marked improvements in liver fibrosis was markedly improved, as evidenced by a decrease in the collagen-stained area in the liver sections. Quantitative analysis indicated that the MCD diet induced a fibrosis index of 17.66%, but concomitantly treatment with butyrate resulted in a 9.54% reduction in fibrosis (*P* < 0.001; **Figure 2C**).

Additionally, we examined the mRNA levels of early pro-fibrogenic markers in the liver. Compared to the MCS diet, the MCD diet significantly increased the expression of *TGF*-β*1* (*P* < 0.05; **Figure 2E**), which is involved in the early stage of fibrosis. Interestingly, butyrate treatment significantly downregulated *TGF*-β*1* expression (*P* < 0.05; **Figure 2E**). Similarly, the MCD diet induced the expression of *Acta2*, a gene encoding alpha smooth muscle actin (α-*SMA*), but the *Acta2* expression level was significantly reduced in mice concomitantly fed butyrate (*P* < 0.001; **Figure 2E**). IHC analysis further confirmed our results: compared to that in the Control group, the protein expression of α-SMA was significantly upregulated in the MCD group (*P* < 0.001; **Figures 2B,D**). Butyrate supplementation significantly reduced the level of α-SMA-positive staining in cells (*P* < 0.01), which was consistent with the Masson’s trichrome staining results (**Figures 2B,D**).

Toll-like receptor (TLR)-4 and its co-receptor CD14 are involved in LPS recognition (Csak et al., 2011). In this study, MCD induced the mRNA expression of endotoxin-associated *TLR4* and *CD14* in the liver (*P* < 0.01 and *P* < 0.001, respectively), and butyrate treatment significantly reversed these effects (*P* < 0.01 and *P* < 0.001, respectively; **Figure 2F**).

### Butyrate Improved the Serum Cytokine Profiles and Reduced the Serum Endotoxin Levels Induced by the MCD Diet

**FIGURE 3:**
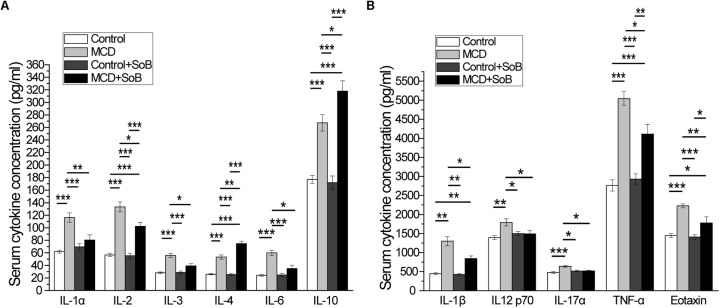
Butyrate improved serum cytokine profiles. **(A,B)** The increased production of pro-inflammatory cytokines IL-1α, IL-1β, IL-2, IL-3, IL-6, IL-12 p70, IL-17α, and TNF-α and the chemokine, eotaxin induced by the MCD diet was significantly attenuated after butyrate treatment, and production of anti-inflammatory cytokines IL-4 and IL-10 was significantly increased. Data are presented as the means ± SEM. *N* = 15–17 per group. ^∗^*P* < 0.05, ^∗∗^*P* < 0.01, and ^∗∗∗^*P* < 0.001 by one-way ANOVA with *post hoc* Tukey’s test.

It has been demonstrated that cytokines, especially pro-inflammatory cytokines, play important roles in the pathological progression of NASH (Tilg and Diehl, 2000), which was evident in the MCD-induced NASH mouse model in this study because of the significant increase in the levels of systemic inflammatory cytokines, including the pro-inflammatory cytokines IL-1α, IL-1β, IL-2, IL-3, IL-6, IL-12 p70, IL-17α, and TNF-α, anti-inflammatory cytokines IL-4 and IL-10, and the chemokine Eotaxin (**Figures 3A,B**). Interestingly, butyrate treatment not only significantly down-regulated the production of pro-inflammatory cytokines (IL-1α, IL-1β, IL-2, IL-3, IL-6, IL-12 p70, IL-17α, TNF-α, and Eotaxin) but also slightly upregulated the production of the anti-inflammatory cytokines IL-4 and IL-10 in the MCD + SoB group relative to that in the MCD group (**Figures 3A,B**). No significant differences were found among the groups in the other cytokines studied.

Lipopolysaccharide acts as a ligand for activating specific TLRs. Therefore, we measured the serum LBP levels to indirectly determine the effect of butyrate treatment on endotoxemia (**Table 1**). Mice in the MCD group displayed significantly higher LBP levels than mice in the Control group (*P* < 0.01). However, endotoxemia was significantly improved in the MCD + SoB group relative to that in the MCD group (*P* < 0.01).

### Supplementation With Butyrate Stabilized the Intestinal Barrier and Regulated Toll-Like Receptors in the Colon

**FIGURE 4:**
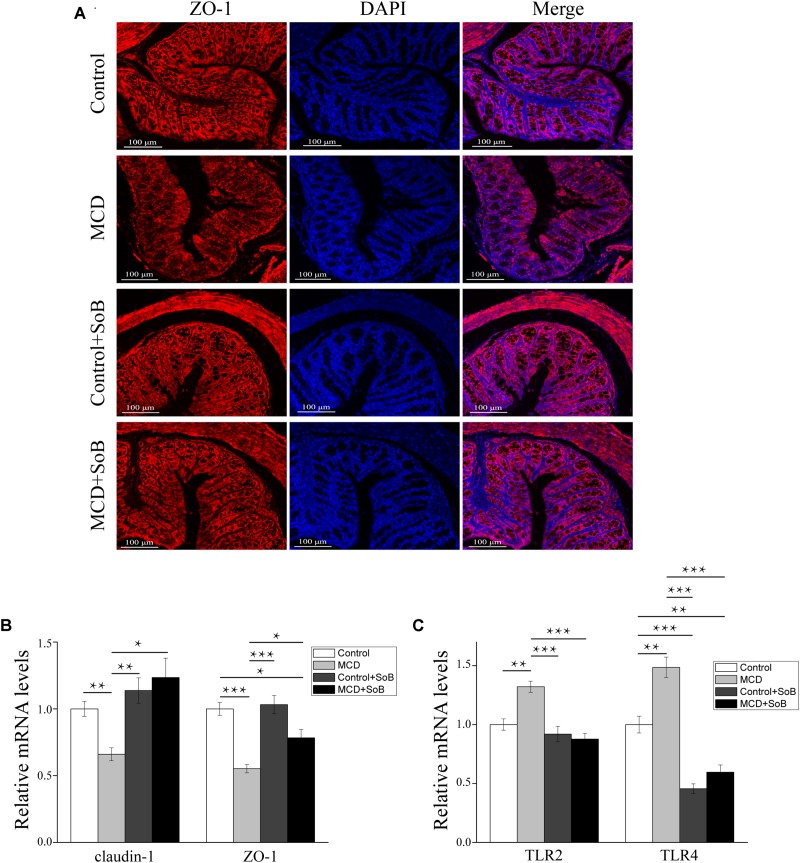
Butyrate improved intestinal barrier function and regulated TLRs in the colon. **(A)** Representative colon histology assessed by ZO-1 immunofluorescence staining (scale bar, 100 μm). **(B,C)** Relative mRNA levels of gut barrier function markers claudin-1 and ZO-1 **(B)** and TLR2 and TLR4 **(C)**. Data are presented as the means ± SEM. *N* = 15–17 per group. ^∗^*P* < 0.05, ^∗∗^*P* < 0.01, and ^∗∗∗^*P* < 0.001 by one-way ANOVA with *post hoc* Tukey’s test *post hoc* ANOVA one-way statistical analysis.

The impairment of intestinal tight junctions (TJs) has been shown to be associated with NASH (Zhu et al., 2013). Therefore, we studied the expression and distribution of TJs proteins in the colon, where the most abundant gut microbiota resides (Wu et al., 2015) and where butyrate is mainly produced and absorbed (Brahe et al., 2013). As depicted in **Figure 4B**, the MCD diet downregulated *claudin-1* and *ZO-1* mRNA levels (*P* < 0.01 and *P* < 0.001, respectively), whereas butyrate treatment reversed this effect and significantly increased *claudin-1* and *ZO-1* expression (*P* < 0.05 and *P* < 0.05, respectively). ZO-1 immunostaining further revealed that the colon tissues in the MCD group had increased disruption and disorganization on the apical surface and in the crypts, but butyrate treatment stabilized TJs structures at the protein level, as evidenced by smooth and organized localization of ZO-1 (**Figure 4A**).

Furthermore, the colon tissue adopted an expression pattern similar to that of the liver, with the MCD diet significantly increasing TLR2 and TLR4 mRNA expression (*P* < 0.01 and *P* < 0.01, respectively); however, these changes in expression levels were strongly suppressed by butyrate supplementation (*P* < 0.001 and *P* < 0.001, respectively; **Figure 4C**).

### Butyrate Treatment Alleviated the Microbiome Dysbiosis Induced by the MCD Diet

To obtain further insights into the protective effects of butyrate, we investigated the impact of butyrate on the gut microbiome using metagenomic sequencing of the 16S rRNA gene. The Simpson index, an estimated species diversity value, was significantly higher in the Control + SoB group than in the Control group (*P* < 0.05; **Supplementary Table S3**). No other significant differences were observed in overall microbial richness and species diversity among the four groups, as estimated by the Chao 1 metric and the Shannon index, respectively (**Supplementary Table S3**).

**FIGURE 5:**
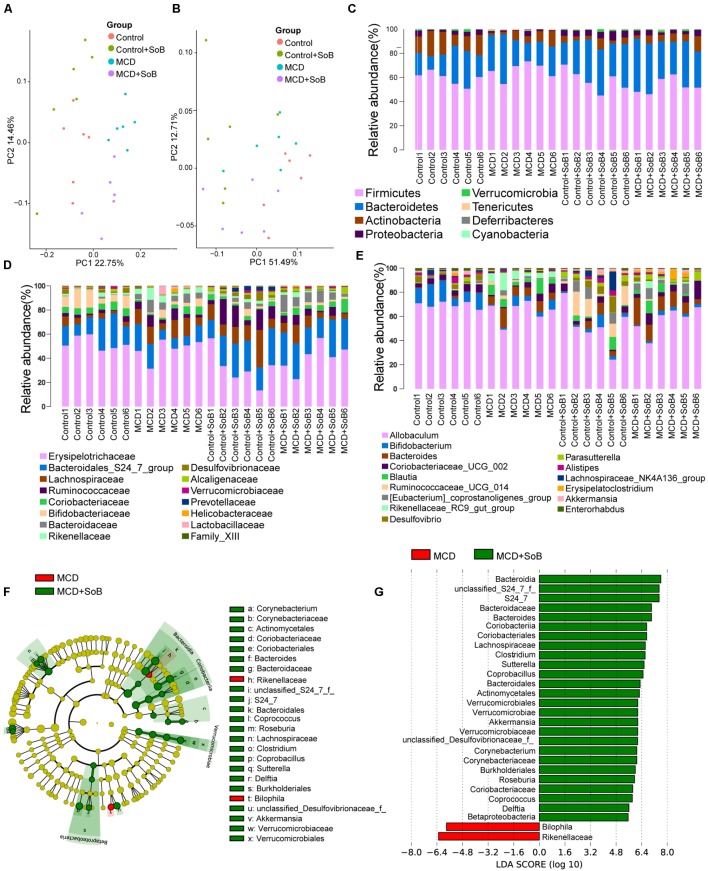
Butyrate alleviated the microbiome dysbiosis induced by the MCD diet. **(A,B)** PCoA plot of the microbiota based on unweighted UniFrac metric **(A)** and weighted UniFrac metric **(B)**. Each symbol represents one sample (*N* = 6 per group). **(C–E)** Top most abundant taxa at the phylum **(C)**, family **(D)**, and genus **(E)** level. **(F)** LEfSe cladogram representing taxon enriched in the MCD (red) and MCD + SoB group (green). Rings from the inside out represented taxonomic levels from phylum to genus levels. Sizes of circles indicate the relative abundance of the taxa. **(G)** Discriminative biomarkers with an LDA score > 4.8.

The unweighted (**Figure 5A**) and weighted (**Figure 5B**) UniFrac principal coordinate analysis (PCoA), which evaluates phylogenetic similarities between microbial communities, was used to calculate the beta-diversity values. The Control group and the MCD group were clearly separated into different clusters (PERMANOVA, unweighted *P* < 0.01, *r* = 0.391; weighted *P* < 0.01, *r* = 0.341). Likewise, the microbiota of the MCD + SoB group was clustered separately from that of the MCD group (PERMANOVA, unweighted *P* < 0.01, *r* = 0.298; weighted *P* < 0.05, *r* = 0.285). Similar results were obtained using the Bray–Curtis distance matrices (**Supplementary Figure S1**).

Despite significant interindividual differences and diverse bacterial communities, the MCD diet with SoB supplementation clearly affected the gut microbial configuration. The top most abundant taxa at the phylum, family, and genus levels are presented in **Figures 5C–E**. Tenericutes was significantly more abundant in the fecal microbiota of the MCD group than in that of the Control group (*P* < 0.01), whereas the Actinobacteria population was significantly reduced in the fecal microbiota of the MCD group (*P* < 0.01). Compared with that of the MCD group, the fecal microbiota of the MCD + SoB group showed a marked decreases in the abundance of Firmicutes and Tenericutes (*P* < 0.01 and *P* < 0.05, respectively), and a significant increase in the abundance of Verrucomicrobia and Proteobacteria were significantly increased (*P* < 0.05 and *P* < 0.05, respectively). At the family level, the relative abundance of *Lachnospiraceae*, *Bacteroidaceae*, and *Rikenellaceae* were significantly higher in the MCD group than in the Control group (*P* < 0.05, *P* < 0.01, and *P* < 0.05, respectively), while the relative abundance of *Bifidobacteriaceae* and *Desulfovibrionaceae* were significantly lower in the MCD group (*P* < 0.05 and *P* < 0.05, respectively). Interestingly, compared with the MCD group, butyrate treatment in the MCD + SoB group significantly restored the abundance of *Rikenellaceae* (*P* < 0.05) and increased the abundance of *Bacteroidales*_S24_7_group, *Alcaligenaceae*, and *Verrucomicrobiaceae* (*P* < 0.01, *P* < 0.05, and *P* < 0.05, respectively). At the genus level, the fecal microbiota of the MCD group had a significantly higher abundance of *Bacteroides*, *Blautia*, and *Rikenellaceae*_RC9_gut_group (*P* < 0.01, *P* < 0.05, and *P* < 0.01, respectively), and a significantly reduced abundance of *Bifidobacterium*, *Desulfovibrio*, and *Enterorhabdus* (*P* < 0.05, *P* < 0.05, and *P* < 0.05, respectively) than the fecal microbiota of the Control group. Compared with the MCD group, butyrate supplementation significantly reduced the abundance of *Rikenellaceae*_RC9_gut_group and *Lachnospiraceae*_NK4A136_group levels in the MCD + SoB group (*P* < 0.05 and *P* < 0.05, respectively) and markedly promoted the abundance of *Parasutterella* and *Akkermansia* (*P* < 0.05 and *P* < 0.05, respectively). To further identify the distinguishing phylotypes in the gut microbiota of the different groups, we performed LEfSe analysis based on the RDP taxonomy data. Compared with the MCD group, butyrate supplementation in the MCD + SoB group partially restored the microbial structure, as evidenced by preponderant *Akkermansia*, *Roseburia*, *Coprococcus*, *Coprobacillus*, *Delftia*, *Corynebacterium*, *Sutterella*, *Bacteroides*, *Clostridium*, and *Coriobacteriaceae* populations (LDA score (log_10_) > 4.8), and reduced *Bilophila* and *Rikenellaceae* abundance (LDA score (-log_10_) > 4.8) (**Figures 5F,G**).

### Butyrate Treatment Extensively Ameliorated the MCD Diet-Induced Fecal Metabolomic Profiles

With an untargeted strategy, we studied the fecal metabolome associated with each functional state of the gut microbiota. A total of 322 metabolites were identified and quantified in the non-targeted metabolomic analysis, covering the following KEGG metabolic pathways: carbohydrates, amino acids, peptides, lipids, nucleotides, energy, xenobiotics, cofactors and vitamins. The following non-metabolic KEGG pathways were also identified: environmental information processing, human diseases, cellular processes, and organismal systems.

**FIGURE 6:**
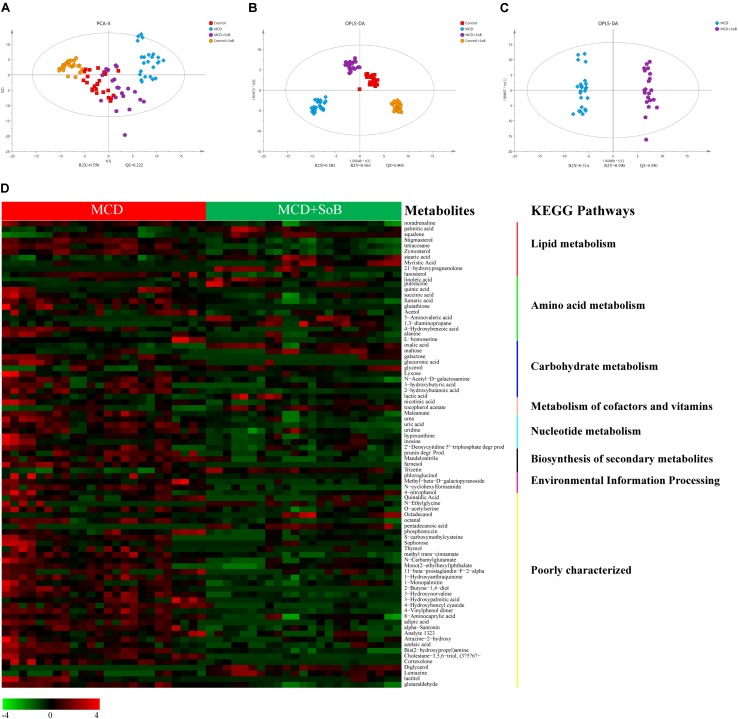
Butyrate extensively ameliorated the changes in fecal metabolomic profile induced by the MCD diet. **(A)** A 4-state model of PCA plot comparing between the Control (red), MCD (blue), Control + SoB (orange), and MCD + SoB (purple) groups. *Q*^2^ (cum) = 0.222 and *R*^2^*X* (cum) = 0.550. **(B)** OPLS-DA score scatter plot comparing between the Control (red), MCD (blue), Control + SoB (orange), and MCD + SoB (purple) groups. *Q*^2^ (cum) = 0.909 and *R*^2^*Y* (cum) = 0.963. **(C)** OPLS-DA score scatter plot comparing the MCD (blue) and MCD + SoB (purple) groups. *Q*^2^ (cum) = 0.950 and *R*^2^*Y* (cum) = 0.990. Each symbol represents one sample (*N* = 24 per group, with a quadruplicate technical replicates of six samples per group, except for the MCD + SoB group, for which three unqualified data set datasets were eliminated). **(D)** Euclidean distance hierarchical clustering analysis demonstrating the different intensity levels of characteristic metabolites.

A PCA was performed (*R*^2^*X* = 0.550, *Q*^2^ = 0.222) using a 4-state model corresponding to the four groups (Control, MCD, Control + SoB, and MCD + SoB), and the score plot (**Figure 6A**) showed a clustering tendency in the direction of the *X*-axis (the first predictive principal component). The MCD + SoB group adopted a configuration closer to that of the Control group. An OPLS-DA using a 4-state model was then performed. As depicted in **Figure 6B**, the score plot of the first predictive principal component (*X*-axis) and first orthogonal principal component (*Y*-axis) showed a significant separation in the metabolomic datasets among the four groups. The cross-validated predictive ability *Q*^2^ was 0.909, indicating that a random fecal GC-MS spectrum discriminates among the four groups at 90.9% of the time. The explained variance *R*^2^ was 0.963. Thus, these results suggest distinct clustering of fecal metabolomic profiles among the groups.

The main focus of the present analysis was to identify differential metabolites that potentially contributed to the separation between the MCD and MCD + SoB groups. Therefore, an OPLS-DA model was established for the two groups (*R*^2^*Y* = 0.990, *Q*^2^ = 0.950) (**Figure 6C**). The characteristic metabolites that were extensively altered following butyrate treatment were selected according to the OPLS-DA model, with VIP values > 1 and *P*-values < 0.05 (**Figure 6D** and **Supplementary Table S4**). Ultimately, 98 metabolites were selected and were mainly associated with pathways involved in the metabolism of lipids, amino acids, carbohydrates, cofactors, vitamins, and nucleotides. Specifically, the abundance of several metabolites involved in lipid metabolic pathways were significantly higher or lower in the MCD + SoB group than those in the MCD group (**Figure 6D**). For example, the levels of saturated fatty acids, such as stearic acid and behenic acid, unsaturated fatty acids, such as oleic acid and linoleic acid, and squalene, a natural 30-carbon triterpene, were significantly increased in the feces of mice in the MCD + SoB group, but the level of arachidonic acid, an unsaturated fatty acid, was significantly decreased by butyrate in this study.

### Butyrate-Modified Gut Microbiota and Fecal Metabolites With Important Roles in NASH Improvement

**FIGURE 7:**
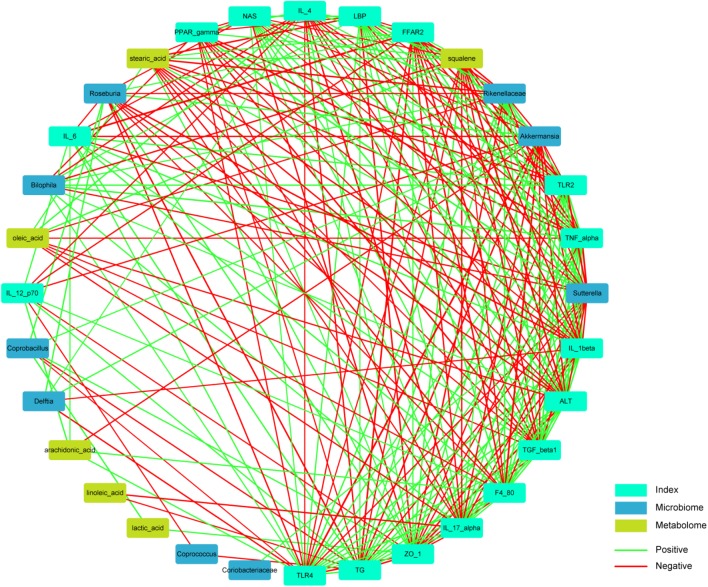
Correlation analysis of representative microbial genera, characterized metabolites, liver, and gut impairment parameters between the MCD and MCD + SoB group. Spearman’s rank correlation was used, and significant associations with *P* < 0.05 and *r* > 0.5 are shown. Green nodes: representative injury parameters; Blue nodes: differentially distributed genera between the MCD group and the MCD + SoB group; Yellow nodes: metabolites with a high discriminative power between the MCD group and the MCD + SoB group. Green lines between nodes indicate positive linkages, and red lines represent negative relationships. The thickness of the connection represents the correlation coefficient, with thicker lines indicating higher *r*-values.

To further elucidate the beneficial role of the altered microbial community, we conducted a correlation analysis of the interaction matrix. The analysis was restricted to the following: representative microbial genera with significantly altered relative abundance between the MCD group and the MCD + SoB group, metabolites with a high discriminative power, and other representative injury parameters (**Figure 7** and **Supplementary Table S5**). *Bilophila* and *Rikenellaceae*, which were clearly increased in the MCD group, may be involved in the inflammatory responses and NASH progression. For example, both *Bilophila* and *Rikenellaceae* were positively correlated with the levels of ALT, IL-17α, LBP, TLR2, TLR4, and TNF-α. Additionally, the abundance of *Bilophila* abundance was highly associated with that of *Rikenellaceae*. In contrast, the relative abundance of several butyrate-promoted probiotic strains may play a role in alleviating liver and intestinal damage. Specifically, *Akkermansia* was significantly negatively correlated with the levels of ALT, NAS, TG, F4/80, IL-12 p70, IL-17α, IL-1β, IL-6, TNF-α, TGF-β1, TLR2, TLR4, and LBP and was positively associated with IL-4 and ZO-1 levels. The enrichment of promising probiotics after butyrate treatment had strong correlations with fecal metabolites. The under-represented arachidonic acid levels in the MCD + SoB group were negatively associated with *Akkermansia*, while over-represented lactic acid levels were positively associated with *Coprobacillus*; squalene and stearic acid levels were positively associated with *Sutterella*. Squalene and stearic acid may improve liver damage, as indicated by the ALT level. Therefore, these data provide evidence that the modified gut microbiota and fecal metabolites induced by butyrate supplementation have important roles in ameliorating NASH.

## Discussion

The major finding of the present study was that supplementation with SoB substantially ameliorated MCD-induced steatohepatitis in mice. Additionally, several complementary mechanisms were identified, including the following: (i) alleviation of microbial dysbiosis; (ii) protection of the intestinal barrier; (iii) improvement of the metabolomic profile; (iv) regulation of lipid metabolism; and (v) attenuation of the inflammatory response.

The liver–gut axis plays a pivotal role in NAFLD pathogenesis and progression (Jiao et al., 2017; Zhu et al., 2017). TLR signaling in hepatic immune cells induces the initial inflammatory response by producing a broad array of cytokines, which may mediate the destruction of the intestinal barrier and subsequent bacterial translocation, and these events can induce secondary TLR signaling activation (Szabo et al., 2010). We found that butyrate treatment downregulated the expression of *TLR4*, the *TLR4* co-receptor *CD14* in the liver, and *TLR2* in the colon (Plovier et al., 2017). It has been demonstrated that *TLR4* signaling may promote NAFLD progression, and NASH is characterized by an increased sensitivity to bacterial LPS, a TLR4 ligand (Szabo et al., 2005). LPS derived from the gut microbiota may be transferred to the liver through the portal circulation, consequently activating TLR4 on Kupffer cells (KCs) and hepatic stellate cells (HSCs) and triggering a cascade of inflammatory signaling pathways, which in turn can induce liver injury and fibrosis (Spruss et al., 2009; Shi et al., 2017).

Kupffer cells have been implicated in NASH development, and in the release of various inflammatory cytokines such as TNF-α, IL-6, and IL-1β upon activation (Bilzer et al., 2006; Cai et al., 2017). These cytokines are known to be involved in liver function impairment (Ni et al., 2016), liver fibrogenesis (Bieghs and Trautwein, 2013), and inflammation-driven dyslipidemia (Nati et al., 2016; Robertson et al., 2017). In this study, hepatic and systemic inflammatory responses were improved in the MCD + SoB group, as evidenced by the decreased expression of pro-inflammatory cytokine genes, such as *IL-1*β, *F4/80*, and *TNF*-α, and increased expression of anti-inflammatory cytokine genes, such as *IL-4*, and *IL-10*, in the liver. Similarly, significantly decreased levels of pro-inflammatory cytokines (IL-1α, IL-1β, IL-2, IL-3, IL-6, IL-12 p70, IL-17α, TNF-α, and eotaxin) and increased production of anti-inflammatory cytokines (IL-4 and IL-10) were observed in the serum. As shown by IHC staining, mice in the MCD + SoB group also displayed with a significant reduction in the number of F4/80^+^ infiltrating macrophages in the liver, which indicated a decreased activation of KCs. Improved liver function and lipid metabolism are indicated by the ALT, AST, and TG levels, which were reduced after butyrate treatment, resulting in the alleviation of both hepatocyte injury and inflammation-driven dyslipidemia.

Hepatic stellate cells serve as another mediator of the liver–gut axis due to their high TLRs expression (Shi et al., 2017). HSCs are key cells that express contractile α-SMA and produce excessive extracellular matrix (ECM) components during liver fibrosis (Yum et al., 2017). Activation of HSCs during liver fibrosis mainly depends on KC secretion of TGF-β1, the most powerful modulator (Wu et al., 2016), and, to a lesser extent, IL-1β (Mridha et al., 2017). The increased expression of TGF-β1 and α-SMA induced by the MCD diet was significantly reduced by butyrate, and the alleviation of fibrosis was further verified by Masson’s trichrome and α-SMA IHC staining.

The ability of butyrate to modulate inflammatory and immune responses, including the suppression of NF-κB activation by inhibiting histone deacetylase (HDAC) (Ye et al., 2017) and to act as a signaling molecule via targeting FFAR-1, 2, and 3 (Mattace Raso et al., 2013; Khan and Jena, 2015) are the most frequently investigated mechanisms related to this SCFA. In energy metabolism, butyrate regulates energy expenditure and mitochondrial function through the promotion of PPAR-γ coactivator PGC-1α, via AMP kinase (AMPK) activation and HDAC inhibition (Mattace Raso et al., 2013). Furthermore, FGF-21, which is involved in the stimulation of fatty acid β-oxidation in the liver, is also induced by butyrate (Li et al., 2012). We found that butyrate could not only increased the expression of FFAR2, PPAR-γ, FGF21, and PGC-1α, which is consistent with previous studies, but may also alter the intestinal microbiota and barrier function to protect the liver from NASH.

The elevated LPS level in NASH patients is mainly due to the impaired intestinal barrier and dysbiosis of the gut microbiota (Zhu et al., 2013; Arab et al., 2017). Consistently, we found that the MCD diet disrupted barrier homeostasis, as evidenced by mRNA expression and immunostaining analyses. The MCD diet favored *Anaeroplasma*, *Bilophila*, *Anaerotruncus*, *Turicibacter*, *SMB53* and *Flexispira*, some species of which are pro-inflammatory bacteria or opportunistic pathogens (Freeman and Holland, 2007; Shen et al., 2016; Horie et al., 2017; Suriano et al., 2017; Zinkernagel et al., 2017). Notably, butyrate shifted the gut microbiota composition by reducing *Bilophila* and *Rikenellaceae* abundance and strongly promoting *Akkermansia*, *Roseburia*, *Coprococcus*, *Coprobacillus*, *Delftia*, *Sutterella*, and *Coriobacteriaceae* genera. Some species of *Bilophila* were identified as LPS-producing bacteria and mucosa-damaging bacteria (Song et al., 2017). Our study consistently found that the abundance of *Bilophila* was positively correlated with LBP levels. Some species in *Rikenellaceae* family have been demonstrated to be pathogenic by augmenting inflammation and the production of mutagenic toxins (Sun et al., 2017), which is consistent with the correlation analysis showing that *Rikenellaceae* abundance was positively associated with F4/80, IL-17α, IL-1β, TGF-β1, and TNF-α levels. Accumulating evidence has confirmed the beneficial roles of the butyrate-producing genera *Akkermansia* and *Roseburia* in alleviating endotoxemia and improving immunological disorders and gut barrier function (Patterson et al., 2017; Wang et al., 2017). We consistently found that both *Akkermansia* and *Roseburia* were inversely correlated with several pro-inflammatory cytokine levels and positively correlated with the gut barrier marker ZO-1. Additionally, *Akkermansia* had a negative association with LBP levels. Our previous study found that *Akkermansia muciniphila* protects against immune-mediated liver injury in a mouse model by alleviating inflammation and hepatocellular death (Wu et al., 2017). The genus *Coprobacillus* and another butyrate-producing genus, *Coprococcus*, have been found to play beneficial roles in maintaining intestinal stability (Arrazuria et al., 2016; Mancabelli et al., 2017; Nishino et al., 2017; Walsh et al., 2017). The probiotic properties of *Delftia* are due to the ability of this genus to transform or degrade multiple organic and inorganic toxins (Li et al., 2017). Furthermore, *Sutterella*, an organism that promotes a protective immunoregulatory profile *in vitro* (Berer et al., 2017), and *Coriobacteriaceae*, some species of which have been suggested to improve host lipid metabolism (Raza et al., 2017), were also increased following butyrate treatment. Therefore, the depletion of opportunistic pathogens and promotion of promising probiotics provide evidence for the beneficial role of butyrate.

To link microbial community structure with metabolic functions, we integrated metabolomic approaches into this study. The over-represented metabolites including stearic acid, behenic acid, oleic acid, linoleic acid, and squalene in the feces of the MCD + SoB group are involved in lipid metabolism pathways for the biosynthesis of unsaturated fatty acids, steroids, and fatty acids, and in the metabolism of linoleic acid. Studies have reported that stearic acid, a potent anti-inflammatory lipid, can exert a protective effect by promoting the recovery of hepatic dysfunction (Hashemi Goradel et al., 2016). Our study also found that stearic acid was negatively correlated with several pro-inflammatory cytokines, ALT, and the NAS. Moreira et al. (2017) suggested that behenic acid results from reduced TG absorption. Although a high concentration of oleic acid is traditionally used to induce steatosis *in vitro* (Porteiro et al., 2017), dietary supplementation with oleic acid has been shown to exert several protective effects associated with energy homeostasis in different experimental models of NAFLD (Lee et al., 2011), especially a hypocholesterolemic effect (Ducheix et al., 2017). Surprisingly, supplementation with an oleic acid-derived compound (S1) restores high fat diet-induced gut microbiota dysbiosis (Mujico et al., 2013). A negative correlation between oleic acid and *Rikenellaceae* was found in our study. Recently, a combination of fatty acids including linoleic acid was found to be associated with a lower incidence of type 2 diabetes (T2D) (Imamura et al., 2017). Squalene is capable of reducing cholesterolemia through the activation of liver X receptor (LXR) α and β without promoting lipogenesis in hepatocytes (Hien et al., 2017). Arachidonic acid, an unsaturated fatty acid that was significantly decreased after butyrate treatment in this study, was positively associated with pro-inflammatory F4/80, IL-12 p70, and IL-6 and inversely correlated with the probiotic *Akkermansia*. Studies have also found that arachidonic acid exacerbates NASH through favoring pro-inflammatory microbiota, reducing butyrate production, amplifying inflammation via the TLR4-NF-κB pathway and inducing insulin resistance (Zhuang et al., 2017). Interestingly, we found that lactic acid was significantly increased in the MCD + SoB group, which indicated cross-feeding between lactate-producing bacteria and lactate-converting bacteria. Butyrate increased the abundance of lactate-producing bacteria, such as *Coprobacillus* (Kageyama and Benno, 2000), in our study. Lactate favors lactate-converting microbiota, such as *Coprococcus* and *Roseburia*, which ultimately produce butyrate (Falony et al., 2009; Flint et al., 2015), constituting a positive feedback loop in the microbiota ecosystem, which substantially amplifies the protective effects.

Nevertheless, our study has some limitations. First, although an MCD dietary model was used as a NASH animal model in this study because morphological changes in the mouse liver resemble human NASH, characterized by hepatic steatosis and inflammatory infiltration (Levada et al., 2018), this model is not entirely representative of human NASH because of the absence of features such as obesity and insulin resistance. Additionally, the metabolic profile of the MCD diet-induced NASH animal model is distinct from that of NASH in humans. Therefore, further clinical studies are needed. Second, our study identified a clear correlation between the butyrate-induced shifts in the gut microbiome and metabolome and alleviated hepatic and intestinal impairment. However, whether the alterations in the gut microbiome and metabolome are the cause or the effect of the hepatic and intestinal improvement requires further investigation.

## Conclusion

Our findings demonstrated that butyrate induced a protective shift in the gut microbiome and metabolome, thereby effectively preventing the MCD diet-induced liver and gut impairments associated with NASH. Thus, the current study indicates that oral butyrate administration is a promising strategy for treating NASH.

## Author Contributions

JY, LXL, WW, YL, and LJL designed the study. JY, WW, and YL collected the data for the study and wrote and edited the manuscript. DS, DF, FG, HJ, RY, and WY contributed to discussions and reviewed the manuscript. All authors read and approved the final manuscript.

## Conflict of Interest Statement

The authors declare that the research was conducted in the absence of any commercial or financial relationships that could be construed as a potential conflict of interest.

## Footnotes

1 http://huttenhower.sph.harvard.edu/galaxy/

## Supplementary Material

FIGURE S1 PCoA plot of the microbiota based on Bray–Curtis distance matrices.

TABLE S1 Detailed ingredient of the MCD diet and MCS diet^1^.

TABLE S2 Specific primers used for the RT-PCR analyses.

TABLE S3 Comparison of diversity estimation of the 16S rRNA gene library at 97% similarity from the pyrosequencing analysis^1^.

TABLE S4 Changes of differential metabolites as determined by GC-MS in the MCD + SoB group compared to the MCD group.

TABLE S5 Significant associations in correlation analysis of representative microbial genera, characterized metabolites, liver, and gut impairment parameters between the MCD and MCD + SoB group^1^.

## References

[B1] Arab J. P. Martin-Mateos R. M. Shah V. H. (2017). Gut-liver axis, cirrhosis and portal hypertension: the chicken and the egg. *Hepatol. Int.* 12(Suppl. 1), 24–33. 10.1007/s12072-017-9798-x 28550391 PMC6876989

[B2] Arrazuria R. Elguezabal N. Juste R. A. Derakhshani H. Khafipour E. (2016). *Mycobacterium avium* subspecies *paratuberculosis* infection modifies gut microbiota under different dietary conditions in a rabbit model. *Front. Microbiol.* 7:446. 10.3389/fmicb.2016.00446 27065994 PMC4815054

[B3] Berer K. Gerdes L. A. Cekanaviciute E. Jia X. Xiao L. Xia Z. (2017). Gut microbiota from multiple sclerosis patients enables spontaneous autoimmune encephalomyelitis in mice. *Proc. Natl. Acad. Sci. U.S.A.* 114 10719–10724. 10.1073/pnas.1711233114 28893994 PMC5635914

[B4] Bieghs V. Trautwein C. (2013). The innate immune response during liver inflammation and metabolic disease. *Trends Immunol.* 34 446–452. 10.1016/j.it.2013.04.005 23668977

[B5] Bilzer M. Roggel F. Gerbes A. L. (2006). Role of Kupffer cells in host defense and liver disease. *Liver Int.* 26 1175–1186. 10.1111/j.1478-3231.2006.01342.x 17105582

[B6] Brahe L. K. Astrup A. Larsen L. H. (2013). Is butyrate the link between diet, intestinal microbiota and obesity-related metabolic diseases? *Obes Rev.* 14 950–959. 10.1111/obr.12068 23947604

[B7] Cai C. Zhu X. Li P. Li J. Gong J. Shen W. (2017). NLRP3 deletion inhibits the non-alcoholic steatohepatitis development and inflammation in Kupffer cells induced by palmitic acid. *Inflammation* 40 1875–1883. 10.1007/s10753-017-0628-z 28730512

[B8] Caporaso J. G. Kuczynski J. Stombaugh J. Bittinger K. Bushman F. D. Costello E. K. (2010). QIIME allows analysis of high-throughput community sequencing data. *Nat. Methods* 7 335–336. 10.1038/nmeth.f.303 20383131 PMC3156573

[B9] Chung C. Y. Alden S. L. Funderburg N. T. Fu P. Levine A. D. (2014). Progressive proximal-to-distal reduction in expression of the tight junction complex in colonic epithelium of virally-suppressed HIV^+^ individuals. *PLoS Pathog.* 10:e1004198. 10.1371/journal.ppat.1004198 24968145 PMC4072797

[B10] Cole J. R. Wang Q. Cardenas E. Fish J. Chai B. Farris R. J. (2009). The ribosomal database project: improved alignments and new tools for rRNA analysis. *Nucleic Acids Res.* 37 D141–D145. 10.1093/nar/gkn879 19004872 PMC2686447

[B11] Csak T. Velayudham A. Hritz I. Petrasek J. Levin I. Lippai D. (2011). Deficiency in myeloid differentiation factor-2 and toll-like receptor 4 expression attenuates nonalcoholic steatohepatitis and fibrosis in mice. *Am. J. Physiol. Gastrointest. Liver Physiol.* 300 G433–G441. 10.1152/ajpgi.00163.2009 21233280 PMC3302188

[B12] Ducheix S. Montagner A. Polizzi A. Lasserre F. Regnier M. Marmugi A. (2017). Dietary oleic acid regulates hepatic lipogenesis through a liver X receptor-dependent signaling. *PLoS One* 12:e0181393. 10.1371/journal.pone.0181393 28732092 PMC5521785

[B13] Falony G. Verschaeren A. De Bruycker F. De Preter V. Verbeke K. Leroy F. (2009). *In vitro* kinetics of prebiotic inulin-type fructan fermentation by butyrate-producing colon bacteria: implementation of online gas chromatography for quantitative analysis of carbon dioxide and hydrogen gas production. *Appl. Environ. Microbiol.* 75 5884–5892. 10.1128/AEM.00876-09 19633122 PMC2747863

[B14] Flint H. J. Duncan S. H. Scott K. P. Louis P. (2015). Links between diet, gut microbiota composition and gut metabolism. *Proc. Nutr. Soc.* 74 13–22. 10.1017/S0029665114001463 25268552

[B15] Freeman A. F. Holland S. M. (2007). Persistent bacterial infections and primary immune disorders. *Curr. Opin. Microbiol.* 10 70–75. 10.1016/j.mib.2006.11.005 17208513

[B16] Gurnell M. (2003). PPARgamma and metabolism: insights from the study of human genetic variants. *Clin. Endocrinol.* 59 267–277. 12919147 10.1046/j.1365-2265.2003.01767.x

[B17] Hashemi Goradel N. Eghbal M. A. Darabi M. Roshangar L. Asadi M. Zarghami N. (2016). Improvement of liver cell therapy in rats by dietary stearic acid. *Iran. Biomed. J.* 20 217–222. 27090202 10.7508/ibj.2016.04.005 PMC4983676

[B18] Hien H. T. M. Ha N. C. Thom L. T. Hong D. D. (2017). Squalene promotes cholesterol homeostasis in macrophage and hepatocyte cells via activation of liver X receptor (LXR) alpha and beta. *Biotechnol. Lett.* 39 1101–1107. 10.1007/s10529-017-2345-y 28492976

[B19] Horie M. Miura T. Hirakata S. Hosoyama A. Sugino S. Umeno A. (2017). Comparative analysis of the intestinal flora in type 2 diabetes and nondiabetic mice. *Exp. Anim.* 66 405–416. 10.1538/expanim.17-0021 28701620 PMC5682353

[B20] Imamura F. Sharp S. J. Koulman A. Schulze M. B. Kroger J. Griffin J. L. (2017). A combination of plasma phospholipid fatty acids and its association with incidence of type 2 diabetes: the EPIC-InterAct case-cohort study. *PLoS Med.* 14:e1002409. 10.1371/journal.pmed.1002409 29020051 PMC5636062

[B21] Jiao N. Baker S. S. Chapa-Rodriguez A. Liu W. Nugent C. A. Tsompana M. (2017). Suppressed hepatic bile acid signalling despite elevated production of primary and secondary bile acids in NAFLD. *Gut* 10.1136/gutjnl-2017-314307 [Epub ahead of print]. 28774887

[B22] Jin C. J. Sellmann C. Engstler A. J. Ziegenhardt D. Bergheim I. (2015). Supplementation of sodium butyrate protects mice from the development of non-alcoholic steatohepatitis (NASH). *Br. J. Nutr.* 114 1745–1755. 10.1017/S0007114515003621 26450277

[B23] Julio Junior H. R. Costa S. F. Costa W. S. Barcellos Sampaio F. J. Favorito L. A. (2017). Structural study of the bladder in fetuses with prune belly syndrome. *Neurourol. Urodyn.* 37 148–152. 10.1002/nau.23327 28598513

[B24] Kageyama A. Benno Y. (2000). *Coprobacillus catenaformis* gen. nov., sp. nov., a new genus and species isolated from human feces. *Microbiol. Immunol.* 44 23–28. 10711596 10.1111/j.1348-0421.2000.tb01242.x

[B25] Keren-Shaul H. Spinrad A. Weiner A. Matcovitch-Natan O. Dvir-Szternfeld R. Ulland T. K. (2017). A unique microglia type associated with restricting development of Alzheimer’s disease. *Cell* 169:e1217. 10.1016/j.cell.2017.05.018 28602351

[B26] Khan S. Jena G. (2015). The role of butyrate, a histone deacetylase inhibitor in diabetes mellitus: experimental evidence for therapeutic intervention. *Epigenomics* 7 669–680. 10.2217/epi.15.20 26111036

[B27] Kleiner D. E. Brunt E. M. Van Natta M. Behling C. Contos M. J. Cummings O. W. (2005). Design and validation of a histological scoring system for nonalcoholic fatty liver disease. *Hepatology* 41 1313–1321. 10.1002/hep.20701 15915461

[B28] Le Roy T. Llopis M. Lepage P. Bruneau A. Rabot S. Bevilacqua C. (2013). Intestinal microbiota determines development of non-alcoholic fatty liver disease in mice. *Gut* 62 1787–1794. 10.1136/gutjnl-2012-303816 23197411

[B29] Lee J. Y. Moon J. H. Park J. S. Lee B. W. Kang E. S. Ahn C. W. (2011). Dietary oleate has beneficial effects on every step of non-alcoholic Fatty liver disease progression in a methionine- and choline-deficient diet-fed animal model. *Diabetes Metab. J.* 35 489–496. 10.4093/dmj.2011.35.5.489 22111040 PMC3221024

[B30] Levada K. Guldiken N. Zhang X. Vella G. Mo F. R. James L. P. (2018). Hsp72 protects from liver injury via attenuation of hepatocellular death, oxidative stress and JNK-signaling. *J. Hepatol.* 68 996–1005. 10.1016/j.jhep.2018.01.003 29331340 PMC9252261

[B31] Li H. Gao Z. Zhang J. Ye X. Xu A. Ye J. (2012). Sodium butyrate stimulates expression of fibroblast growth factor 21 in liver by inhibition of histone deacetylase 3. *Diabetes Metab. Res. Rev.* 61 797–806. 10.2337/db11-0846 22338096 PMC3314370

[B32] Li O. Xiao R. Sun L. Guan C. Kong D. Hu X. (2017). Bacterial and diazotrophic diversities of endophytes in *Dendrobium catenatum* determined through barcoded pyrosequencing. *PLoS One* 12:e0184717. 10.1371/journal.pone.0184717 28931073 PMC5607135

[B33] Liu R. Hong J. Xu X. Feng Q. Zhang D. Gu Y. (2017). Gut microbiome and serum metabolome alterations in obesity and after weight-loss intervention. *Nat. Med.* 23 859–868. 10.1038/nm.4358 28628112

[B34] Loomba R. Sirlin C. B. Ang B. Bettencourt R. Jain R. Salotti J. (2015). Ezetimibe for the treatment of nonalcoholic steatohepatitis: assessment by novel magnetic resonance imaging and magnetic resonance elastography in a randomized trial (MOZART trial). *Hepatology* 61 1239–1250. 10.1002/hep.27647 25482832 PMC4407930

[B35] Lu H. Ren Z. Li A. Zhang H. Jiang J. Xu S. (2016). Deep sequencing reveals microbiota dysbiosis of tongue coat in patients with liver carcinoma. *Sci. Rep.* 6:33142. 10.1038/srep33142 27605161 PMC5015078

[B36] Mancabelli L. Milani C. Lugli G. A. Turroni F. Mangifesta M. Viappiani A. (2017). Unveiling the gut microbiota composition and functionality associated with constipation through metagenomic analyses. *Sci. Rep.* 7:9879. 10.1038/s41598-017-10663-w 28852182 PMC5575163

[B37] Mattace Raso G. Simeoli R. Russo R. Iacono A. Santoro A. Paciello O. (2013). Effects of sodium butyrate and its synthetic amide derivative on liver inflammation and glucose tolerance in an animal model of steatosis induced by high fat diet. *PLoS One* 8:e68626. 10.1371/journal.pone.0068626 23861927 PMC3702592

[B38] Moreira D. K. Santos P. S. Gambero A. Macedo G. A. (2017). Evaluation of structured lipids with behenic acid in the prevention of obesity. *Food Res. Int.* 95 52–58. 10.1016/j.foodres.2017.03.005 28395825

[B39] Mridha A. R. Wree A. Robertson A. A. B. Yeh M. M. Johnson C. D. Van Rooyen D. M. (2017). NLRP3 inflammasome blockade reduces liver inflammation and fibrosis in experimental NASH in mice. *J. Hepatol.* 66 1037–1046. 10.1016/j.jhep.2017.01.022 28167322 PMC6536116

[B40] Mujico J. R. Baccan G. C. Gheorghe A. Diaz L. E. Marcos A. (2013). Changes in gut microbiota due to supplemented fatty acids in diet-induced obese mice. *Br. J. Nutr.* 110 711–720. 10.1017/S0007114512005612 23302605

[B41] Nati M. Haddad D. Birkenfeld A. L. Koch C. A. Chavakis T. Chatzigeorgiou A. (2016). The role of immune cells in metabolism-related liver inflammation and development of non-alcoholic steatohepatitis (NASH). *Rev. Endocr. Metab. Disord.* 17 29–39. 10.1007/s11154-016-9339-2 26847547

[B42] Ni Y. Zhuge F. Nagashimada M. Ota T. (2016). Novel action of carotenoids on non-alcoholic fatty liver disease: macrophage polarization and liver homeostasis. *Nutrients* 8:E391. 10.3390/nu8070391 27347998 PMC4963867

[B43] Nishino K. Nishida A. Inoue R. Kawada Y. Ohno M. Sakai S. (2017). Analysis of endoscopic brush samples identified mucosa-associated dysbiosis in inflammatory bowel disease. *J. Gastroenterol.* 53 95–106. 10.1007/s00535-017-1384-4 28852861

[B44] Patterson A. M. Mulder I. E. Travis A. J. Lan A. Cerf-Bensussan N. Gaboriau-Routhiau V. (2017). Human gut symbiont *Roseburia hominis* promotes and regulates innate immunity. *Front. Immunol.* 8:1166. 10.3389/fimmu.2017.01166 29018440 PMC5622956

[B45] Plovier H. Everard A. Druart C. Depommier C. Van Hul M. Geurts L. (2017). A purified membrane protein from *Akkermansia muciniphila* or the pasteurized bacterium improves metabolism in obese and diabetic mice. *Nat. Med.* 23 107–113. 10.1038/nm.4236 27892954

[B46] Porteiro B. Fondevila M. F. Delgado T. C. Iglesias C. Imbernon M. Iruzubieta P. (2017). Hepatic p63 regulates steatosis via IKKbeta/ER stress. *Nat. Commun.* 8:15111. 10.1038/ncomms15111 28480888 PMC5424198

[B47] Raza G. S. Putaala H. Hibberd A. A. Alhoniemi E. Tiihonen K. Makela K. A. (2017). Polydextrose changes the gut microbiome and attenuates fasting triglyceride and cholesterol levels in Western diet fed mice. *Sci. Rep.* 7:5294. 10.1038/s41598-017-05259-3 28706193 PMC5509720

[B48] Robertson J. Porter D. Sattar N. Packard C. J. Caslake M. McInnes I. (2017). Interleukin-6 blockade raises LDL via reduced catabolism rather than via increased synthesis: a cytokine-specific mechanism for cholesterol changes in rheumatoid arthritis. *Ann. Rheum. Dis.* 76 1949–1952. 10.1136/annrheumdis-2017-211708 28916714

[B49] Rosso C. Mezzabotta L. Gaggini M. Salomone F. Gambino R. Marengo A. (2016). Peripheral insulin resistance predicts liver damage in nondiabetic subjects with nonalcoholic fatty liver disease. *Hepatology* 63 107–116. 10.1002/hep.28287 26473614

[B50] Shen H. Lu Z. Chen Z. Wu Y. Shen Z. (2016). Rapid fermentable substance modulates interactions between ruminal commensals and toll-like receptors in promotion of immune tolerance of goat rumen. *Front. Microbiol.* 7:1812. 10.3389/fmicb.2016.01812 27909428 PMC5112275

[B51] Shi D. Lv L. Fang D. Wu W. Hu C. Xu L. (2017). Administration of *Lactobacillus salivarius* LI01 or *Pediococcus pentosaceus* LI05 prevents CCl4-induced liver cirrhosis by protecting the intestinal barrier in rats. *Sci. Rep.* 7:6927. 10.1038/s41598-017-07091-1 28761060 PMC5537250

[B52] Song J. J. Tian W. J. Kwok L. Y. Wang Y. L. Shang Y. N. Menghe B. (2017). Effects of microencapsulated *Lactobacillus plantarum* LIP-1 on the gut microbiota of hyperlipidaemic rats. *Br. J. Nutr.* 118 481–492. 10.1017/S0007114517002380 29017628

[B53] Spruss A. Kanuri G. Wagnerberger S. Haub S. Bischoff S. C. Bergheim I. (2009). Toll-like receptor 4 is involved in the development of fructose-induced hepatic steatosis in mice. *Hepatology* 50 1094–1104. 10.1002/hep.23122 19637282

[B54] Sun T. Liu S. Zhou Y. Yao Z. Zhang D. Cao S. (2017). Evolutionary biologic changes of gut microbiota in an ‘adenoma-carcinoma sequence’ mouse colorectal cancer model induced by 1, 2-Dimethylhydrazine. *Oncotarget* 8 444–457. 10.18632/oncotarget.13443 27880935 PMC5352133

[B55] Suriano F. Bindels L. B. Verspreet J. Courtin C. M. Verbeke K. Cani P. D. (2017). Fat binding capacity and modulation of the gut microbiota both determine the effect of wheat bran fractions on adiposity. *Sci. Rep.* 7:5621. 10.1038/s41598-017-05698-y 28717237 PMC5514075

[B56] Szabo G. Billiar T. R. Machida K. Crispe I. N. Seki E. (2010). Toll-like receptor signaling in liver diseases. *Gastroenterol. Res. Pract.* 2010:971270. 10.1155/2010/971270 21789039 PMC3123973

[B57] Szabo G. Velayudham A. Romics L. Jr. Mandrekar P. (2005). Modulation of non-alcoholic steatohepatitis by pattern recognition receptors in mice: the role of toll-like receptors 2 and 4. *Alcohol. Clin. Exp. Res.* 29 140S–145S. 16344599 10.1097/01.alc.0000189287.83544.33

[B58] Tilg H. Diehl A. M. (2000). Cytokines in alcoholic and nonalcoholic steatohepatitis. *N. Engl. J. Med.* 343 1467–1476. 10.1056/NEJM200011163432007 11078773

[B59] Tremellen K. McPhee N. Pearce K. (2017). Metabolic endotoxaemia related inflammation is associated with hypogonadism in overweight men. *Basic Clin. Androl.* 27:5. 10.1186/s12610-017-0049-8 28286655 PMC5341351

[B60] Walsh C. J. Guinane C. M. O’Toole P. W. Cotter P. D. (2017). A profile hidden markov model to investigate the distribution and frequency of LanB-encoding lantibiotic modification genes in the human oral and gut microbiome. *PeerJ* 5:e3254. 10.7717/peerj.3254 28462050 PMC5410138

[B61] Wang J. H. Bose S. Lim S. K. Ansari A. Chin Y. W. Choi H. S. (2017). *Houttuynia cordata* facilitates metformin on ameliorating insulin resistance associated with gut microbiota alteration in OLETF rats. *Genes* 8:E239. 10.3390/genes8100239 28937612 PMC5664089

[B62] Wu S. Yi J. Zhang Y. G. Zhou J. Sun J. (2015). Leaky intestine and impaired microbiome in an amyotrophic lateral sclerosis mouse model. *Physiol. Rep.* 3:e12356. 10.14814/phy2.12356 25847918 PMC4425962

[B63] Wu W. Lv L. Shi D. Ye J. Fang D. Guo F. (2017). Protective effect of *Akkermansia muciniphila* against immune-mediated liver injury in a mouse model. *Front. Microbiol.* 8:1804. 10.3389/fmicb.2017.01804 29033903 PMC5626943

[B64] Wu X. Ma Y. Shao F. Tan Y. Tan T. Gu L. (2016). CUG-binding protein 1 regulates HSC activation and liver fibrogenesis. *Nat. Commun.* 7:13498. 10.1038/ncomms13498 27853137 PMC5118555

[B65] Ye J. Wu W. Li Y. Li L. (2017). Influences of the gut microbiota on DNA methylation and histone modification. *Dig. Dis. Sci.* 62 1155–1164. 10.1007/s10620-017-4538-6 28341870

[B66] Yum M. J. Koppula S. Kim J. S. Shin G. M. Chae Y. J. Yoon T. (2017). Protective effects of *Ampelopsis brevipedunculata* against *in vitro* hepatic stellate cells system and thioacetamide-induced liver fibrosis rat model. *Pharm. Biol.* 55 1577–1585. 10.1080/13880209.2017.1311928 28395572 PMC6130492

[B67] Zhou D. Pan Q. Xin F. Z. Zhang R. N. He C. X. Chen G. Y. (2017). Sodium butyrate attenuates high-fat diet-induced steatohepatitis in mice by improving gut microbiota and gastrointestinal barrier. *World J. Gastroenterol.* 23 60–75. 10.3748/wjg.v23.i1.60 28104981 PMC5221287

[B68] Zhu L. Baker R. D. Zhu R. Baker S. S. (2017). Sequencing the gut metagenome as a noninvasive diagnosis for advanced nonalcoholic steatohepatitis. *Hepatology* 66 2080–2083. 10.1002/hep.29387 28732121

[B69] Zhu L. Baker S. S. Gill C. Liu W. Alkhouri R. Baker R. D. (2013). Characterization of gut microbiomes in nonalcoholic steatohepatitis (NASH) patients: a connection between endogenous alcohol and NASH. *Hepatology* 57 601–609. 10.1002/hep.26093 23055155

[B70] Zhuang P. Shou Q. Lu Y. Wang G. Qiu J. Wang J. (2017). Arachidonic acid sex-dependently affects obesity through linking gut microbiota-driven inflammation to hypothalamus-adipose-liver axis. *Biochim. Biophys. Acta* 1863 2715–2726. 10.1016/j.bbadis.2017.07.003 28711599

[B71] Zinkernagel M. S. Zysset-Burri D. C. Keller I. Berger L. E. Leichtle A. B. Largiader C. R. (2017). Association of the intestinal microbiome with the development of neovascular age-related macular degeneration. *Sci. Rep.* 7:40826. 10.1038/srep40826 28094305 PMC5240106

