# Supplementary figures and images for: Butyrate Protects Mice Against Methionine–Choline-Deficient Diet-Induced Non-alcoholic Steatohepatitis by Improving Gut Barrier Function, Attenuating Inflammation and Reducing Endotoxin Levels
Source: Front Microbiol. 2018 Aug 21;9:1967. doi: 10.3389/fmicb.2018.01967 (PMC6111843; doi:10.3389/fmicb.2018.01967)

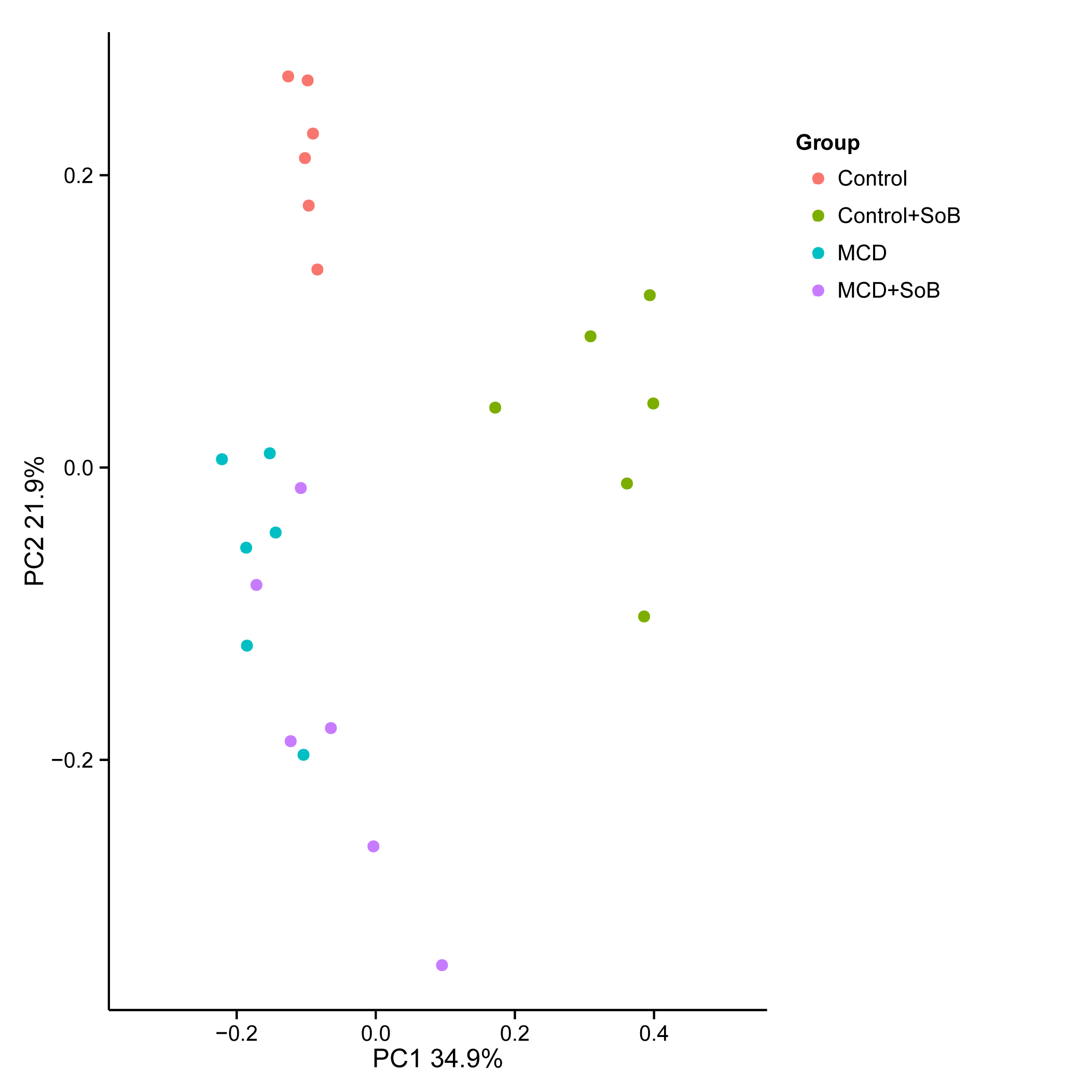

Supplement: FIGURE S1 — PCoA plot of the microbiota based on Bray–Curtis distance matrices. [file Image_1.TIF]
