# Supplementary material for: Butyrate Protects Mice Against Methionine–Choline-Deficient Diet-Induced Non-alcoholic Steatohepatitis by Improving Gut Barrier Function, Attenuating Inflammation and Reducing Endotoxin Levels
Source: Front Microbiol. 2018 Aug 21;9:1967. doi: 10.3389/fmicb.2018.01967 (PMC6111843; doi:10.3389/fmicb.2018.01967)
Supplement: TABLE S1 — Detailed ingredient of the MCD diet and MCS diet1. [file Table_1.docx]

Supplementary Material

Butyrate protects mice against methionine-choline-deficient diet-induced nonalcoholic steatohepatitis by improving gut barrier function, attenuating inflammation and reducing endotoxin levels

Jianzhong Ye, Longxian Lv, Wenrui Wu, Yating Li, Ding Shi, Daiqiong Fang, Feifei Guo, Huiyong Jiang, Ren Yan, Wanchun Ye, Lanjuan Li*

*** Correspondence:** Lanjuan Li: ljli@zju.edu.cn

# Supplementary Table S1 Detailed ingredient of the MCD diet and MCS diet^1^.

| **Diet** | **MCD diet** | | **MCS diet** | |
| --- | --- | --- | --- | --- |
|  | **gm** | **kcal** | **gm** | **kcal** |
| Protein | 17 | 16 | 17 | 16 |
| Carbohydrate | 66 | 63 | 65 | 62 |
| Fat | 10 | 21 | 10 | 21 |
|  |  | 100 |  | 100 |
|  | 4.2 |  | 4.2 |  |
| Ingredient (gm) |  |  |  |  |
| L-Alanine | 3.5 | 14 | 3.5 | 14 |
| L-Arginine | 12.1 | 48.4 | 12.1 | 48.4 |
| L-Asparagine-H2O | 6 | 24 | 6 | 24 |
| L-Aspartate | 3.5 | 14 | 3.5 | 14 |
| L-Cystine | 3.5 | 14 | 3.5 | 14 |
| L-Glutamine | 40 | 160 | 40 | 160 |
| Glycine | 23.3 | 93.2 | 23.3 | 93.2 |
| L-Histidine-HCl-H2O | 4.5 | 18 | 4.5 | 18 |
| L-Isoleucine | 8.2 | 32.8 | 8.2 | 32.8 |
| L-Leucine | 11.1 | 44.4 | 11.1 | 44.4 |
| L-Lysine-HCl | 18 | 72 | 18 | 72 |
| L-Phenylalanine | 7.5 | 30 | 7.5 | 30 |
| L-Proline | 3.5 | 14 | 3.5 | 14 |
| L-Serine | 3.5 | 14 | 3.5 | 14 |
| L-Threonine | 8.2 | 32.8 | 8.2 | 32.8 |
| L-Tryptophan | 1.8 | 7.2 | 1.8 | 7.2 |
| L-Tyrosine | 5 | 20 | 5 | 20 |
| L-Valine | 8.2 | 32.8 | 8.2 | 32.8 |
| Total L-Amino Acids | 171.4 | 685.6 | 171.4 | 685.6 |
| Sucrose | 455.3 | 1821.2 | 452.3 | 1809.2 |
| Corn Starch | 150 | 600 | 150 | 600 |
| Maltodextrin 10 | 50 | 200 | 50 | 200 |
| Cellulose | 30 | 0 | 30 | 0 |
| Corn Oil | 100 | 900 | 100 | 900 |
| Mineral Mix S10001 | 35 | 0 | 35 | 0 |
| Sodium Bicarbonate | 7.5 | 0 | 7.5 | 0 |
| Vitamin Mix V10001 | 10 | 40 | 10 | 40 |
| L-Methionine | 0 | 0 | 3 | 12 |
| Choline Bitrartrate | 0 | 0 | 2 | 0 |
| Total | 1009.2 | 4246.8 | 1011.2 | 4246.8 |

# ^1^MCD: methionine-choline deficient; MCS: methionine-choline sufficient
