# Supplementary material for: Butyrate Protects Mice Against Methionine–Choline-Deficient Diet-Induced Non-alcoholic Steatohepatitis by Improving Gut Barrier Function, Attenuating Inflammation and Reducing Endotoxin Levels
Source: Front Microbiol. 2018 Aug 21;9:1967. doi: 10.3389/fmicb.2018.01967 (PMC6111843; doi:10.3389/fmicb.2018.01967)
Supplement: TABLE S2 — Specific primers used for the RT-PCR analyses. [file Table_2.docx]

Supplementary Material

Butyrate protects mice against methionine-choline-deficient diet-induced nonalcoholic steatohepatitis by improving gut barrier function, attenuating inflammation and reducing endotoxin levels

Jianzhong Ye, Longxian Lv, Wenrui Wu, Yating Li, Ding Shi, Daiqiong Fang, Feifei Guo, Huiyong Jiang, Ren Yan, Wanchun Ye, Lanjuan Li*

*** Correspondence:** Lanjuan Li: ljli@zju.edu.cn

# Supplementary Table S2 Specific primers used for the RT-PCR analyses.

| **Gene** | **Forward Sequence (5 ' - 3 ' )** | **Reverse Sequence (5' - 3 ' )** | **Product Length (bp)** |
| --- | --- | --- | --- |
| IL-1β | GAAATGCCACCTTTTGACAGTG | TGGATGCTCTCATCAGGACAG | 116 |
| IL-4 | GGTCTCAACCCCCAGCTAGT | GCCGATGATCTCTCTCAAGTGAT | 102 |
| IL-10 | CTTACTGACTGGCATGAGGATCA | GCAGCTCTAGGAGCATGTGG | 101 |
| F4/80 | TGACTCACCTTGTGGTCCTAA | CTTCCCAGAATCCAGTCTTTCC | 111 |
| PPAR-γ | GGAAGACCACTCGCATTCCTT | GTAATCAGCAACCATTGGGTCA | 121 |
| FGF21 | GTGTCAAAGCCTCTAGGTTTCTT | GGTACACATTGTAACCGTCCTC | 123 |
| PGC1-α | TATGGAGTGACATAGAGTGTGCT | GTCGCTACACCACTTCAATCC | 143 |
| FFAR2 | CTTGATCCTCACGGCCTACAT | CCAGGGTCAGATTAAGCAGGAG | 137 |
| TGF-β1 | CTTCAATACGTCAGACATTCGGG | GTAACGCCAGGAATTGTTGCTA | 142 |
| Acta2 | GGCACCACTGAACCCTAAGG | ACAATACCAGTTGTACGTCCAGA | 135 |
| TLR2 | CACCACTGCCCGTAGATGAAG | AGGGTACAGTCGTCGAACTCT | 148 |
| TLR4 | ATGGCATGGCTTACACCACC | GAGGCCAATTTTGTCTCCACA | 129 |
| CD14 | ACTTCTCAGATCCGAAGCCAG | CCGCCGTACAATTCCACAT | 76 |
| claudin-1 | TGCCCCAGTGGAAGATTTACT | CTTTGCGAAACGCAGGACAT | 94 |
| ZO-1 | GCCGCTAAGAGCACAGCAA | GCCCTCCTTTTAACACATCAGA | 172 |
