# Supplementary material for: Butyrate Protects Mice Against Methionine–Choline-Deficient Diet-Induced Non-alcoholic Steatohepatitis by Improving Gut Barrier Function, Attenuating Inflammation and Reducing Endotoxin Levels
Source: Front Microbiol. 2018 Aug 21;9:1967. doi: 10.3389/fmicb.2018.01967 (PMC6111843; doi:10.3389/fmicb.2018.01967)
Supplement: TABLE S3 — Comparison of diversity estimation of the 16S rRNA gene library at 97% similarity from the pyrosequencing analysis1. [file Table_3.docx]

Supplementary Material

Butyrate protects mice against methionine-choline-deficient diet-induced nonalcoholic steatohepatitis by improving gut barrier function, attenuating inflammation and reducing endotoxin levels

Jianzhong Ye, Longxian Lv, Wenrui Wu, Yating Li, Ding Shi, Daiqiong Fang, Feifei Guo, Huiyong Jiang, Ren Yan, Wanchun Ye, Lanjuan Li*

*** Correspondence:** Lanjuan Li: ljli@zju.edu.cn

# Supplementary Table S3 Comparison of diversity estimation of the 16S rRNA gene library at 97% similarity from the pyrosequencing analysis^1^.

| **Diversity index** | **Control** | **MCD** | **Control+SoB** | **MCD+SoB** |
| --- | --- | --- | --- | --- |
| Chao1 | 2321±225 | 2726±464 | 2583±107 | 2671±151 |
| Shannon | 5.401±0.196 | 5.636±0.193 | 5.976±0.268 | 5.600±0.154 |
| Simpson | 0.916±0.007 | 0.920±0.010 | 0.954±0.011* | 0.940±0.008 |

^1^All data are given as the means ± SEM. *P < 0.05 vs. the Control group.
