# Supplementary material for: Butyrate Protects Mice Against Methionine–Choline-Deficient Diet-Induced Non-alcoholic Steatohepatitis by Improving Gut Barrier Function, Attenuating Inflammation and Reducing Endotoxin Levels
Source: Front Microbiol. 2018 Aug 21;9:1967. doi: 10.3389/fmicb.2018.01967 (PMC6111843; doi:10.3389/fmicb.2018.01967)
Supplement: TABLE S4 — Changes of differential metabolites as determined by GC-MS in the MCD + SoB group compared to the MCD group. [file Table_4.docx]

Supplementary Material

Butyrate protects mice against methionine-choline-deficient diet-induced nonalcoholic steatohepatitis by improving gut barrier function, attenuating inflammation and reducing endotoxin levels

Jianzhong Ye, Longxian Lv, Wenrui Wu, Yating Li, Ding Shi, Daiqiong Fang, Feifei Guo, Huiyong Jiang, Ren Yan, Wanchun Ye, Lanjuan Li*

*** Correspondence:** Lanjuan Li: ljli@zju.edu.cn

# Supplementary Table S4 Changes of differential metabolites as determined by GC-MS in the MCD+SoB group compared to the MCD group.

| **Metabolites** | **MZ** | **RT(min)** | **VIP** | **P-value** | **Fold change** | **Average (MCD+SoB)** | **Average (MCD)** |
| --- | --- | --- | --- | --- | --- | --- | --- |
| oxalic acid | 147 | 5.4855 | 2.1166 | 3.36E-07 | 1.97846528 | 290.662356 | 146.913044 |
| 1-Hydroxyanthraquinone | 121 | 5.5308 | 1.8159 | 7.00E-08 | 0.51533884 | 8.211379 | 15.933943 |
| lactic acid | 117 | 5.6811 | 1.0198 | 0.03066775 | 1.42763270 | 50.702648 | 35.515191 |
| farnesol | 107 | 5.8253 | 2.5730 | 7.85E-10 | 0.00000002 | 0.000001 | 55.645912 |
| succinate semialdehyde | 89 | 5.8535 | 1.4450 | 0.02477486 | 3.58419930 | 0.641903 | 0.179093 |
| tetracosane | 71 | 5.8780 | 2.1009 | 3.30E-11 | 0.54255405 | 250.399815 | 461.520497 |
| hexadecane | 71 | 5.9218 | 1.0542 | 0.00070986 | 0.67680144 | 69.513263 | 102.708503 |
| 3-Methylthiopropylamine | 174 | 5.9367 | 1.0274 | 3.01E-06 | 0.47824569 | 2.966234 | 6.202322 |
| Nicotinoylglycine | 207 | 5.9567 | 1.0502 | 0.00274013 | 0.74665660 | 15.032438 | 20.133000 |
| 2-hydroxybutanoic acid | 131 | 6.0970 | 2.1774 | 3.57E-10 | 0.28863289 | 4.538751 | 15.724995 |
| glutathione | 107 | 6.5289 | 1.7517 | 0.00022775 | 0.00000150 | 0.000001 | 0.667818 |
| Maleamate | 151 | 7.0473 | 1.3776 | 6.36E-06 | 0.53897135 | 6.300858 | 11.690525 |
| 3-Hydroxypyridine | 152 | 7.5545 | 1.0335 | 0.00217371 | 0.72244938 | 1.861695 | 2.576922 |
| adrenaline | 267 | 7.7118 | 1.1315 | 0.01118885 | 0.20719441 | 7.060615 | 34.077247 |
| methyl trans-cinnamate | 131 | 7.7364 | 1.1500 | 2.40E-10 | 0.31217077 | 1.015266 | 3.252279 |
| 1,3-diaminopropane | 70 | 8.0907 | 1.6142 | 1.13E-05 | 4.61151010 | 3.740079 | 0.811031 |
| Gallic acid | 281 | 8.1650 | 1.3937 | 3.28E-06 | 1.58315412 | 19.511038 | 12.324156 |
| N-cyclohexylformamide | 57 | 8.4043 | 1.8491 | 1.48E-10 | 0.14244502 | 0.707222 | 4.964878 |
| 2-Butyne-1,4-diol | 107 | 8.5585 | 2.5759 | 2.66E-10 | 0.00007647 | 0.000184 | 2.406737 |
| N-Ethylglycine | 71 | 8.7023 | 1.5665 | 0.02966977 | 2.49665595 | 1.926213 | 0.771517 |
| 4-Vinylphenol dimer | 192 | 9.3630 | 1.8615 | 2.28E-08 | 0.57992084 | 9.920344 | 17.106377 |
| glycerol | 205 | 9.5843 | 1.4316 | 0.00030822 | 1.90081637 | 1057.876623 | 556.538044 |
| 3-Hydroxynorvaline | 131 | 9.7159 | 1.9476 | 1.70E-10 | 0.35185558 | 17.502928 | 49.744636 |
| nicotinic acid | 106 | 9.7510 | 1.1605 | 0.00511832 | 0.71412897 | 3.226703 | 4.518375 |
| phloroglucinol | 73 | 10.0905 | 1.2093 | 0.00490482 | 0.12677237 | 8.733065 | 68.887764 |
| Methyl-beta-D-galactopyranoside | 204 | 10.1783 | 1.7142 | 8.78E-05 | 0.23027556 | 0.977998 | 4.247076 |
| urea | 189 | 10.3571 | 1.5502 | 6.59E-06 | 0.51624276 | 0.401248 | 0.777247 |
| fumaric acid | 80 | 10.4658 | 1.8593 | 1.12E-11 | 0.40227358 | 0.782746 | 1.945806 |
| Thymol | 172 | 10.6620 | 1.5231 | 0.00106473 | 0.18498685 | 0.030176 | 0.163127 |
| Analyte 420 | 72 | 10.8260 | 1.0971 | 0.02401369 | 0.00001406 | 0.000001 | 0.071131 |
| 2-amino-3-(4-hydroxyphenyl)propanoic acid | 194 | 10.8520 | 1.1658 | 0.00170237 | 0.04085060 | 0.001706 | 0.041765 |
| Analyte 476 | 191 | 11.6304 | 1.4143 | 1.72E-05 | 0.15120421 | 0.042366 | 0.280189 |
| L-homoserine | 218 | 11.7557 | 1.1253 | 0.00036814 | 0.29529223 | 0.489047 | 1.656146 |
| 2-amino-2-methylpropane-1,3-diol | 218 | 11.9008 | 1.0922 | 0.02245882 | 2.28534886 | 0.816805 | 0.357409 |
| phosphomycin | 72 | 12.1299 | 1.0175 | 9.30E-05 | 0.35259748 | 0.963518 | 2.732629 |
| Aminomalonic acid | 218 | 12.1720 | 1.4582 | 0.00034086 | 2.47646444 | 0.911963 | 0.368252 |
| O-acetylserine | 116 | 12.5115 | 1.5057 | 1.29E-05 | 0.73440073 | 1.038904 | 1.414628 |
| Acetol | 217 | 13.5899 | 1.1051 | 0.00452602 | 0.50810691 | 0.419084 | 0.824795 |
| Dioctyl phthalate | 149 | 13.5979 | 1.0255 | 0.00214059 | 0.80478347 | 4.559149 | 5.665063 |
| 3-aminopropionitrile | 71 | 13.6156 | 1.5757 | 1.67E-06 | 0.61541425 | 3.306223 | 5.372354 |
| 4-Hydroxybenzoic acid | 193 | 13.9769 | 1.3773 | 1.16E-05 | 0.22044585 | 0.079544 | 0.360834 |
| 5-Aminovaleric acid | 174 | 14.0236 | 1.8149 | 3.00E-05 | 7.46797863 | 249.828991 | 33.453362 |
| 3-hydroxyphenylacetic acid | 179 | 14.1189 | 1.4127 | 0.00216933 | 2.61250964 | 4.508226 | 1.725630 |
| Lyxose | 103 | 14.4378 | 1.4168 | 0.01061327 | 0.36089230 | 8.893190 | 24.642227 |
| heptadecanoic acid | 117 | 14.9693 | 1.1747 | 7.72E-06 | 1.64361850 | 724.853428 | 441.010749 |
| Diglycerol | 207 | 15.2439 | 2.3717 | 1.28E-13 | 49.53738490 | 12.866102 | 0.259725 |
| azelaic acid | 201 | 15.8578 | 1.0072 | 0.00078656 | 0.39322354 | 0.399326 | 1.015519 |
| hypoxanthine | 206 | 16.0571 | 1.4529 | 0.00036678 | 0.52104973 | 0.529775 | 1.016745 |
| Atrazine-2-hydroxy | 194 | 16.3882 | 1.1750 | 0.0088246 | 0.41675708 | 0.142426 | 0.341749 |
| myo-inositol | 217 | 17.0089 | 1.1727 | 0.00668979 | 2.18871429 | 7.700011 | 3.518052 |
| palmitic acid | 117 | 17.2305 | 1.3568 | 0.00036813 | 1.27072984 | 1012.454304 | 796.750238 |
| Arachidic acid | 117 | 17.3245 | 1.6752 | 2.51E-08 | 1.86548858 | 428.996134 | 229.964492 |
| galactose | 205 | 17.4111 | 1.4820 | 0.00037839 | 0.26224379 | 3.228828 | 12.312313 |
| D-Talose | 205 | 17.5072 | 1.3193 | 0.00014669 | 1.82080391 | 188.025799 | 103.265265 |
| glucuronic acid | 160 | 18.0372 | 2.3503 | 2.21E-07 | 0.08333749 | 0.046646 | 0.559724 |
| lactitol | 75 | 18.3484 | 1.2466 | 0.00802621 | 0.19109730 | 0.246164 | 1.288159 |
| 21-hydroxypregnenolone | 157 | 18.4729 | 1.3658 | 0.00017453 | 774533.07400000 | 0.774533 | 0.000001 |
| octanal | 86 | 18.7705 | 1.7452 | 4.90E-08 | 0.69542059 | 14.603731 | 20.999854 |
| Quinaldic Acid | 143 | 19.2984 | 1.1220 | 0.00284074 | 0.43308145 | 0.474423 | 1.095459 |
| 4-Hydroxybenzyl cyanide | 190 | 19.4664 | 1.6545 | 1.18E-08 | 0.17028280 | 0.054999 | 0.322986 |
| N-Carbamylglutamate | 174 | 20.2381 | 1.1760 | 4.38E-05 | 0.43993023 | 1.585170 | 3.603231 |
| N-Acetyl-D-galactosamine | 71 | 20.4806 | 1.6359 | 1.47E-07 | 0.71367879 | 45.016231 | 63.076319 |
| Indolelactate | 202 | 21.4542 | 1.5480 | 0.00013995 | 0.00000226 | 0.000001 | 0.442558 |
| stearic acid | 117 | 22.3049 | 1.5170 | 1.16E-05 | 1.19804043 | 689.229122 | 575.297050 |
| Nonanoic acid methyl ester | 87 | 22.4152 | 1.0795 | 0.00494611 | 1.29110777 | 6.607043 | 5.117344 |
| linoleic acid | 67 | 22.5189 | 1.0490 | 0.01292189 | 1.80434980 | 67.629813 | 37.481542 |
| adipic acid | 185 | 22.5362 | 1.6108 | 1.04E-07 | 0.12231240 | 0.125801 | 1.028523 |
| Mandelonitrile | 190 | 22.6017 | 1.3358 | 1.03E-06 | 0.36640115 | 0.291516 | 0.795620 |
| Analyte 1323 | 163 | 22.6753 | 1.4809 | 0.00349378 | 0.48080565 | 0.330422 | 0.687225 |
| Bis(2-hydroxypropyl)amine | 160 | 23.0678 | 1.0643 | 6.03E-09 | 0.38787074 | 0.369734 | 0.953241 |
| arachidonic acid | 80 | 24.3193 | 1.0307 | 0.00523506 | 0.52847822 | 15.727713 | 29.760381 |
| oleic acid | 129 | 25.1285 | 1.4051 | 3.15E-05 | 1.92159195 | 251.725451 | 130.998390 |
| N-Acetyl-beta-alanine | 158 | 25.1701 | 1.3876 | 0.00016361 | 0.32900940 | 1.648511 | 5.010527 |
| 11-beta-prostaglandin-F-2-alpha | 191 | 25.7426 | 1.0327 | 0.00989797 | 0.40374258 | 0.057848 | 0.143280 |
| pentadecanoic acid | 117 | 26.7585 | 2.0894 | 6.75E-07 | 7.30715155 | 0.908751 | 0.124365 |
| Tricetin | 233 | 27.1313 | 1.5877 | 4.39E-06 | 1.64865621 | 31.648966 | 19.196826 |
| inosine | 209 | 27.6709 | 1.0217 | 0.04394455 | 0.57025932 | 3.364458 | 5.899874 |
| Elaidic acid | 129 | 28.5240 | 1.2396 | 0.00107667 | 0.00000036 | 0.000001 | 2.756662 |
| Behenic acid | 132 | 28.9407 | 1.0472 | 0.00020935 | 1.57585324 | 63.602946 | 40.360958 |
| 2-Monoolein | 219 | 29.2730 | 1.0597 | 0.00729816 | 0.31923522 | 0.081281 | 0.254612 |
| squalene | 81 | 32.0570 | 1.5655 | 1.76E-06 | 1.53781604 | 53.839632 | 35.010450 |
| beta-Glycerophosphoric acid | 243 | 34.0617 | 1.0282 | 0.04308868 | 1.57895105 | 0.342918 | 0.217181 |
| 5-Methylresorcinol | 253 | 35.8210 | 1.0913 | 0.03285804 | 0.07056420 | 0.011809 | 0.167349 |
| tocopherol acetate | 165 | 36.9500 | 1.4800 | 3.33E-05 | 1.99157524 | 40.580898 | 20.376282 |
| Cholestane-3,5,6-triol | 195 | 37.0944 | 2.3895 | 3.01E-10 | 0.15257404 | 1.336520 | 8.759813 |
| cholesterol | 129 | 37.4286 | 1.5861 | 9.11E-06 | 0.71974612 | 405.797082 | 563.805863 |
| uridine | 139 | 38.0338 | 1.7061 | 0.00024311 | 0.79647393 | 3.337294 | 4.190086 |
| Zymosterol | 129 | 38.9886 | 1.9874 | 2.98E-10 | 0.60877354 | 144.564955 | 237.469183 |
| Stigmasterol | 83 | 39.3923 | 1.9188 | 1.13E-10 | 0.54532211 | 45.245106 | 82.969505 |
| Lumazine | 309 | 39.8400 | 1.4503 | 9.74E-05 | 2.49829344 | 3.792452 | 1.518017 |
| ?Sitosterol | 129 | 40.3672 | 1.6156 | 7.60E-07 | 0.63911640 | 389.698895 | 609.746351 |
| quinic acid | 255 | 40.7702 | 1.4623 | 0.00061022 | 0.00721875 | 0.006177 | 0.855689 |
| uric acid | 57 | 41.4543 | 2.0268 | 1.05E-12 | 0.32667370 | 16.781417 | 51.370579 |
| 3-Hydroxypalmitic acid | 195 | 41.9440 | 1.7131 | 4.95E-06 | 0.07916528 | 0.114876 | 1.451097 |
| alpha-Santonin | 204 | 42.4715 | 1.9986 | 1.49E-08 | 0.56982164 | 0.633708 | 1.112117 |
| lanosterol | 75 | 42.9494 | 2.1637 | 8.89E-09 | 0.54055815 | 5.037417 | 9.318918 |
| Cortexolone | 177 | 43.9697 | 2.1835 | 8.55E-08 | 0.04622574 | 0.042791 | 0.925699 |
| prunin degr. Prod. | 219 | 45.3037 | 1.3960 | 4.14E-06 | 0.20425889 | 0.089815 | 0.439713 |
