# Supplementary material for: Butyrate Protects Mice Against Methionine–Choline-Deficient Diet-Induced Non-alcoholic Steatohepatitis by Improving Gut Barrier Function, Attenuating Inflammation and Reducing Endotoxin Levels
Source: Front Microbiol. 2018 Aug 21;9:1967. doi: 10.3389/fmicb.2018.01967 (PMC6111843; doi:10.3389/fmicb.2018.01967)
Supplement: TABLE S5 — Significant associations in correlation analysis of representative microbial genera, characterized metabolites, liver, and gut impairment parameters between the MCD and MCD + SoB group1. [file Table_5.docx]

Supplementary Material

Butyrate protects mice against methionine-choline-deficient diet-induced nonalcoholic steatohepatitis by improving gut barrier function, attenuating inflammation and reducing endotoxin levels

Jianzhong Ye, Longxian Lv, Wenrui Wu, Yating Li, Ding Shi, Daiqiong Fang, Feifei Guo, Huiyong Jiang, Ren Yan, Wanchun Ye, Lanjuan Li*

*** Correspondence:** Lanjuan Li: ljli@zju.edu.cn

# Supplementary Table S5 Significant associations in correlation analysis of representative microbial genera, characterized metabolites, liver and gut impairment parameters between the MCD and MCD+SoB group^1^.

| **Taxa** | **Env** | **Correlation** | **P value** | **Significance** |
| --- | --- | --- | --- | --- |
| Coprococcus | IL_12_p70 | -0.61538 | 0.037335 | * |
| Coprococcus | TG | -0.65734 | 0.023981 | * |
| Rikenellaceae | Bilophila | 0.615385 | 0.037335 | * |
| Rikenellaceae | Sutterella | -0.6993 | 0.014539 | * |
| Rikenellaceae | Roseburia | -0.6233 | 0.030358 | * |
| Rikenellaceae | Akkermansia | -0.69231 | 0.015878 | * |
| Rikenellaceae | squalene | -0.8042 | 0.002746 | ** |
| Rikenellaceae | stearic_acid | -0.81119 | 0.002369 | ** |
| Rikenellaceae | oleic_acid | -0.60839 | 0.040002 | * |
| Rikenellaceae | IL_1beta | 0.727273 | 0.010001 | * |
| Rikenellaceae | F4_80 | 0.811189 | 0.002369 | ** |
| Rikenellaceae | TNF_alpha | 0.664336 | 0.022159 | * |
| Rikenellaceae | TGF_beta1 | 0.664336 | 0.022159 | * |
| Rikenellaceae | TLR4 | 0.741259 | 0.008171 | ** |
| Rikenellaceae | ZO_1 | -0.79021 | 0.003617 | ** |
| Rikenellaceae | FFAR2 | -0.70629 | 0.013286 | * |
| Rikenellaceae | TLR2 | 0.748252 | 0.007353 | ** |
| Rikenellaceae | IL_17_alpha | 0.615385 | 0.037335 | * |
| Rikenellaceae | ALT | 0.781087 | 0.002705 | ** |
| Rikenellaceae | TG | 0.657343 | 0.023981 | * |
| Rikenellaceae | LBP | 0.811189 | 0.002369 | ** |
| Rikenellaceae | NAS | 0.576821 | 0.04959 | * |
| Bilophila | Sutterella | -0.68531 | 0.017305 | * |
| Bilophila | squalene | -0.72727 | 0.010001 | * |
| Bilophila | TNF_alpha | 0.776224 | 0.00466 | ** |
| Bilophila | IL_4 | -0.68531 | 0.017305 | * |
| Bilophila | TLR4 | 0.615385 | 0.037335 | * |
| Bilophila | PPAR_gamma | -0.67832 | 0.018825 | * |
| Bilophila | TLR2 | 0.727273 | 0.010001 | * |
| Bilophila | IL_17_alpha | 0.692308 | 0.015878 | * |
| Bilophila | ALT | 0.609458 | 0.035395 | * |
| Bilophila | LBP | 0.636364 | 0.030114 | * |
| Coprobacillus | Roseburia | 0.76628 | 0.003653 | ** |
| Coprobacillus | Akkermansia | 0.601844 | 0.038407 | * |
| Coprobacillus | lactic_acid | 0.754118 | 0.004604 | ** |
| Coprobacillus | TLR4 | -0.58009 | 0.048012 | * |
| Coprobacillus | ZO_1 | 0.674356 | 0.016163 | * |
| Coprobacillus | TG | -0.68886 | 0.013229 | * |
| Delftia | Roseburia | 0.757479 | 0.004324 | ** |
| Delftia | Akkermansia | 0.66283 | 0.018816 | * |
| Delftia | IL_1beta | -0.62384 | 0.030172 | * |
| Delftia | IL_4 | 0.577052 | 0.049477 | * |
| Delftia | TLR4 | -0.74861 | 0.005091 | ** |
| Sutterella | Coriobacteriaceae | 0.601399 | 0.042807 | * |
| Sutterella | squalene | 0.825175 | 0.001719 | ** |
| Sutterella | stearic_acid | 0.685315 | 0.017305 | * |
| Sutterella | IL_1beta | -0.73427 | 0.009052 | ** |
| Sutterella | F4_80 | -0.65035 | 0.025912 | * |
| Sutterella | TNF_alpha | -0.92308 | 0 | *** |
| Sutterella | IL_4 | 0.671329 | 0.020442 | * |
| Sutterella | TGF_beta1 | -0.90909 | 0 | *** |
| Sutterella | TLR4 | -0.62238 | 0.0348 | * |
| Sutterella | PPAR_gamma | 0.846154 | 0.00097 | *** |
| Sutterella | ZO_1 | 0.643357 | 0.027955 | * |
| Sutterella | FFAR2 | 0.776224 | 0.00466 | ** |
| Sutterella | TLR2 | -0.91608 | 0 | *** |
| Sutterella | IL_6 | -0.67133 | 0.020442 | * |
| Sutterella | IL_17_alpha | -0.6993 | 0.014539 | * |
| Sutterella | ALT | -0.71454 | 0.009024 | ** |
| Sutterella | TG | -0.6993 | 0.014539 | * |
| Sutterella | LBP | -0.73427 | 0.009052 | ** |
| Sutterella | NAS | -0.57682 | 0.04959 | * |
| Roseburia | Akkermansia | 0.724072 | 0.007751 | ** |
| Roseburia | IL_1beta | -0.78752 | 0.002358 | ** |
| Roseburia | F4_80 | -0.59344 | 0.041938 | * |
| Roseburia | TGF_beta1 | -0.60464 | 0.037281 | * |
| Roseburia | TLR4 | -0.76886 | 0.003472 | ** |
| Roseburia | ZO_1 | 0.709143 | 0.00981 | ** |
| Roseburia | FFAR2 | 0.630764 | 0.027866 | * |
| Roseburia | IL_6 | -0.59717 | 0.040343 | * |
| Roseburia | ALT | -0.7403 | 0.005899 | ** |
| Roseburia | TG | -0.78006 | 0.002764 | ** |
| Roseburia | NAS | -0.58755 | 0.044545 | * |
| Akkermansia | arachidonic_acid | -0.65035 | 0.025912 | * |
| Akkermansia | IL_1beta | -0.72727 | 0.010001 | * |
| Akkermansia | F4_80 | -0.8951 | 5.94E-06 | *** |
| Akkermansia | TNF_alpha | -0.64336 | 0.027955 | * |
| Akkermansia | IL_4 | 0.601399 | 0.042807 | * |
| Akkermansia | TGF_beta1 | -0.73427 | 0.009052 | ** |
| Akkermansia | TLR4 | -0.9021 | 0 | *** |
| Akkermansia | ZO_1 | 0.762238 | 0.005897 | ** |
| Akkermansia | FFAR2 | 0.818182 | 0.002027 | ** |
| Akkermansia | TLR2 | -0.62238 | 0.0348 | * |
| Akkermansia | IL_6 | -0.76923 | 0.005253 | ** |
| Akkermansia | IL_12_p70 | -0.65035 | 0.025912 | * |
| Akkermansia | IL_17_alpha | -0.72727 | 0.010001 | * |
| Akkermansia | ALT | -0.71804 | 0.008539 | ** |
| Akkermansia | TG | -0.81818 | 0.002027 | ** |
| Akkermansia | LBP | -0.59441 | 0.045753 | * |
| Akkermansia | NAS | -0.63714 | 0.025858 | * |
| squalene | stearic_acid | 0.699301 | 0.014539 | * |
| squalene | oleic_acid | 0.727273 | 0.010001 | * |
| squalene | IL_1beta | -0.71329 | 0.012114 | * |
| squalene | F4_80 | -0.6014 | 0.042807 | * |
| squalene | TNF_alpha | -0.81818 | 0.002027 | ** |
| squalene | IL_4 | 0.734266 | 0.009052 | ** |
| squalene | TGF_beta1 | -0.76923 | 0.005253 | ** |
| squalene | TLR4 | -0.61538 | 0.037335 | * |
| squalene | PPAR_gamma | 0.699301 | 0.014539 | * |
| squalene | ZO_1 | 0.755245 | 0.006597 | ** |
| squalene | FFAR2 | 0.608392 | 0.040002 | * |
| squalene | TLR2 | -0.74825 | 0.007353 | ** |
| squalene | IL_17_alpha | -0.74825 | 0.007353 | ** |
| squalene | ALT | -0.85114 | 0.000446 | *** |
| squalene | TG | -0.65734 | 0.023981 | * |
| squalene | LBP | -0.65734 | 0.023981 | * |
| squalene | NAS | -0.57682 | 0.04959 | * |
| stearic_acid | oleic_acid | 0.643357 | 0.027955 | * |
| stearic_acid | IL_1beta | -0.71329 | 0.012114 | * |
| stearic_acid | F4_80 | -0.72028 | 0.011021 | * |
| stearic_acid | TNF_alpha | -0.67133 | 0.020442 | * |
| stearic_acid | TGF_beta1 | -0.64336 | 0.027955 | * |
| stearic_acid | ZO_1 | 0.685315 | 0.017305 | * |
| stearic_acid | FFAR2 | 0.622378 | 0.0348 | * |
| stearic_acid | TLR2 | -0.72727 | 0.010001 | * |
| stearic_acid | IL_17_alpha | -0.60839 | 0.040002 | * |
| stearic_acid | ALT | -0.76357 | 0.00385 | ** |
| stearic_acid | LBP | -0.81818 | 0.002027 | ** |
| stearic_acid | NAS | -0.59567 | 0.040979 | * |
| oleic_acid | F4_80 | -0.6014 | 0.042807 | * |
| oleic_acid | TNF_alpha | -0.60839 | 0.040002 | * |
| oleic_acid | IL_4 | 0.741259 | 0.008171 | ** |
| oleic_acid | TGF_beta1 | -0.58741 | 0.048845 | * |
| oleic_acid | IL_17_alpha | -0.65035 | 0.025912 | * |
| oleic_acid | ALT | -0.64799 | 0.022686 | * |
| arachidonic_acid | F4_80 | 0.65035 | 0.025912 | * |
| arachidonic_acid | TLR4 | 0.692308 | 0.015878 | * |
| arachidonic_acid | IL_6 | 0.622378 | 0.0348 | * |
| arachidonic_acid | IL_12_p70 | 0.72028 | 0.011021 | * |
| linoleic_acid | IL_17_alpha | -0.71329 | 0.012114 | * |
| linoleic_acid | TG | -0.62937 | 0.032395 | * |
| lactic_acid | ZO_1 | 0.664336 | 0.022159 | * |
| IL_1beta | F4_80 | 0.79021 | 0.003617 | ** |
| IL_1beta | TNF_alpha | 0.699301 | 0.014539 | * |
| IL_1beta | IL_4 | -0.65035 | 0.025912 | * |
| IL_1beta | TGF_beta1 | 0.797203 | 0.003161 | ** |
| IL_1beta | TLR4 | 0.748252 | 0.007353 | ** |
| IL_1beta | PPAR_gamma | -0.6014 | 0.042807 | * |
| IL_1beta | ZO_1 | -0.7972 | 0.003161 | ** |
| IL_1beta | FFAR2 | -0.76923 | 0.005253 | ** |
| IL_1beta | TLR2 | 0.846154 | 0.00097 | *** |
| IL_1beta | IL_6 | 0.734266 | 0.009052 | ** |
| IL_1beta | IL_17_alpha | 0.601399 | 0.042807 | * |
| IL_1beta | ALT | 0.795098 | 0.001993 | ** |
| IL_1beta | TG | 0.734266 | 0.009052 | ** |
| IL_1beta | LBP | 0.657343 | 0.023981 | * |
| IL_1beta | NAS | 0.659762 | 0.019573 | * |
| F4_80 | TNF_alpha | 0.734266 | 0.009052 | ** |
| F4_80 | TGF_beta1 | 0.762238 | 0.005897 | ** |
| F4_80 | TLR4 | 0.846154 | 0.00097 | *** |
| F4_80 | ZO_1 | -0.76224 | 0.005897 | ** |
| F4_80 | FFAR2 | -0.8042 | 0.002746 | ** |
| F4_80 | TLR2 | 0.769231 | 0.005253 | ** |
| F4_80 | IL_6 | 0.762238 | 0.005897 | ** |
| F4_80 | IL_12_p70 | 0.587413 | 0.048845 | * |
| F4_80 | IL_17_alpha | 0.657343 | 0.023981 | * |
| F4_80 | ALT | 0.728548 | 0.007202 | ** |
| F4_80 | TG | 0.657343 | 0.023981 | * |
| F4_80 | LBP | 0.755245 | 0.006597 | ** |
| F4_80 | NAS | 0.701233 | 0.011053 | * |
| TNF_alpha | IL_4 | -0.69231 | 0.015878 | * |
| TNF_alpha | TGF_beta1 | 0.895105 | 5.94E-06 | *** |
| TNF_alpha | TLR4 | 0.664336 | 0.022159 | * |
| TNF_alpha | PPAR_gamma | -0.88811 | 9.17E-05 | *** |
| TNF_alpha | ZO_1 | -0.67133 | 0.020442 | * |
| TNF_alpha | FFAR2 | -0.70629 | 0.013286 | * |
| TNF_alpha | TLR2 | 0.881119 | 0.000192 | *** |
| TNF_alpha | IL_17_alpha | 0.797203 | 0.003161 | ** |
| TNF_alpha | ALT | 0.777584 | 0.00291 | ** |
| TNF_alpha | TG | 0.622378 | 0.0348 | * |
| TNF_alpha | LBP | 0.755245 | 0.006597 | ** |
| TNF_alpha | NAS | 0.746474 | 0.00529 | ** |
| IL_4 | TGF_beta1 | -0.58741 | 0.048845 | * |
| IL_4 | TLR4 | -0.59441 | 0.045753 | * |
| IL_4 | PPAR_gamma | 0.65035 | 0.025912 | * |
| IL_4 | ZO_1 | 0.615385 | 0.037335 | * |
| IL_4 | FFAR2 | 0.692308 | 0.015878 | * |
| IL_4 | TLR2 | -0.67133 | 0.020442 | * |
| IL_4 | IL_6 | -0.61538 | 0.037335 | * |
| IL_4 | IL_12_p70 | -0.62937 | 0.032395 | * |
| IL_4 | IL_17_alpha | -0.78322 | 0.004115 | ** |
| IL_4 | ALT | -0.60596 | 0.036759 | * |
| IL_4 | TG | -0.72028 | 0.011021 | * |
| TGF_beta1 | TLR4 | 0.699301 | 0.014539 | * |
| TGF_beta1 | PPAR_gamma | -0.79021 | 0.003617 | ** |
| TGF_beta1 | ZO_1 | -0.62238 | 0.0348 | * |
| TGF_beta1 | FFAR2 | -0.77622 | 0.00466 | ** |
| TGF_beta1 | TLR2 | 0.846154 | 0.00097 | *** |
| TGF_beta1 | IL_6 | 0.699301 | 0.014539 | * |
| TGF_beta1 | IL_17_alpha | 0.692308 | 0.015878 | * |
| TGF_beta1 | ALT | 0.763574 | 0.00385 | ** |
| TGF_beta1 | TG | 0.664336 | 0.022159 | * |
| TGF_beta1 | LBP | 0.622378 | 0.0348 | * |
| TGF_beta1 | NAS | 0.689923 | 0.01303 | * |
| TLR4 | PPAR_gamma | -0.65734 | 0.023981 | * |
| TLR4 | ZO_1 | -0.78322 | 0.004115 | ** |
| TLR4 | FFAR2 | -0.70629 | 0.013286 | * |
| TLR4 | TLR2 | 0.671329 | 0.020442 | * |
| TLR4 | IL_6 | 0.699301 | 0.014539 | * |
| TLR4 | IL_17_alpha | 0.594406 | 0.045753 | * |
| TLR4 | ALT | 0.728548 | 0.007202 | ** |
| TLR4 | TG | 0.741259 | 0.008171 | ** |
| TLR4 | LBP | 0.685315 | 0.017305 | * |
| TLR4 | NAS | 0.633372 | 0.027032 | * |
| PPAR_gamma | ZO_1 | 0.643357 | 0.027955 | * |
| PPAR_gamma | TLR2 | -0.74825 | 0.007353 | ** |
| PPAR_gamma | IL_17_alpha | -0.61538 | 0.037335 | * |
| PPAR_gamma | ALT | -0.64448 | 0.023677 | * |
| PPAR_gamma | TG | -0.60839 | 0.040002 | * |
| PPAR_gamma | LBP | -0.60839 | 0.040002 | * |
| PPAR_gamma | NAS | -0.64468 | 0.023621 | * |
| ZO_1 | FFAR2 | 0.643357 | 0.027955 | * |
| ZO_1 | TLR2 | -0.67832 | 0.018825 | * |
| ZO_1 | IL_12_p70 | -0.64336 | 0.027955 | * |
| ZO_1 | IL_17_alpha | -0.65734 | 0.023981 | * |
| ZO_1 | ALT | -0.86515 | 0.000279 | *** |
| ZO_1 | TG | -0.79021 | 0.003617 | ** |
| ZO_1 | LBP | -0.8042 | 0.002746 | ** |
| ZO_1 | NAS | -0.73139 | 0.006868 | ** |
| FFAR2 | TLR2 | -0.84615 | 0.00097 | *** |
| FFAR2 | IL_6 | -0.93706 | 0 | *** |
| FFAR2 | IL_12_p70 | -0.65035 | 0.025912 | * |
| FFAR2 | IL_17_alpha | -0.76224 | 0.005897 | ** |
| FFAR2 | ALT | -0.57793 | 0.049049 | * |
| FFAR2 | TG | -0.88112 | 0.000192 | *** |
| FFAR2 | LBP | -0.6014 | 0.042807 | * |
| TLR2 | IL_6 | 0.762238 | 0.005897 | ** |
| TLR2 | IL_17_alpha | 0.678322 | 0.018825 | * |
| TLR2 | ALT | 0.669003 | 0.017359 | * |
| TLR2 | TG | 0.699301 | 0.014539 | * |
| TLR2 | LBP | 0.741259 | 0.008171 | ** |
| TLR2 | NAS | 0.618292 | 0.032116 | * |
| IL_6 | IL_12_p70 | 0.685315 | 0.017305 | * |
| IL_6 | TG | 0.769231 | 0.005253 | ** |
| IL_12_p70 | TG | 0.678322 | 0.018825 | * |
| IL_17_alpha | ALT | 0.763574 | 0.00385 | ** |
| IL_17_alpha | TG | 0.79021 | 0.003617 | ** |
| IL_17_alpha | LBP | 0.615385 | 0.037335 | * |
| IL_17_alpha | NAS | 0.625832 | 0.029495 | * |
| ALT | TG | 0.679511 | 0.01507 | * |
| ALT | LBP | 0.7986 | 0.00184 | ** |
| ALT | NAS | 0.823316 | 0.001 | *** |
| TG | LBP | 0.594406 | 0.045753 | * |
| LBP | NAS | 0.712543 | 0.009309 | ** |

^1^ **P* < 0.05, ***P* < 0.01, and ****P* < 0.001.
